# Axonal Domain Structure as a Putative Identifier of Neuron-Specific Vulnerability to Oxidative Stress in Cultured Neurons

**DOI:** 10.1523/ENEURO.0139-22.2022

**Published:** 2022-10-24

**Authors:** Samuel Burke, Louis-Eric Trudeau

**Affiliations:** 1 Department of Pharmacology and Physiology, Faculty of Medicine, Université de Montréal, Montreal, Quebec, H3T 1J4, Canada; 2 Department of Neurosciences, Faculty of Medicine, Université de Montréal, Montreal, Quebec, H3T 1J4, Canada; 3 Neural Signaling and Circuitry Research Group (SNC), Montréal, Quebec, H3T 1J4, Canada; 4 Center for Interdisciplinary Research on the Brain and Learning (CIRCA), Montréal, Quebec, H3T 1J4.

**Keywords:** axon branching, axon terminals, dopamine, Parkinson’s, vulnerability

## Abstract

Several populations of neurons are purported to degenerate in Parkinson’s disease (PD). One current hypothesis suggests that vulnerable neurons in PD share common characteristics including projecting to voluminous territories and having extremely long and branched axonal domains with large numbers of neurotransmitter release sites. In this study, we used a mouse *in vitro* culture system to compare the axonal domain of neuronal populations suspected to be vulnerable in PD to that of neuronal populations considered at a lesser risk. In the first category, we included dopamine (DA) neurons of the substantia nigra, noradrenergic neurons of the locus coeruleus (LC), serotonin neurons of the raphe nuclei (R), and cholinergic neurons of the dorsal motor nucleus of the vagus (DMV). In the second category, we included DA neurons of the ventral tegmental area, cholinergic neurons of the hypoglossal nucleus, and cholinergic interneurons of the dorsal striatum. Validating their differential vulnerability, we find that, when compared with neurons presumed to be resilient in PD, a larger proportion of neurons presumed to be vulnerable in PD degenerate in response to cell stress induced by hydrogen peroxide. We also find that they are endowed with larger axonal domains, that are more complex, have more axonal varicosities with a higher proportion of varicosities that are positive for synaptotagmin 1 (Syt-1). Notwithstanding the obvious limitations related to the dissection of small brain nuclei and to the growth of these neurons *in vitro*, these findings support the hypothesis that axonal domain structure is a key characteristic of neuronal vulnerability to oxidative stress.

## Visual Abstract

**Figure:**
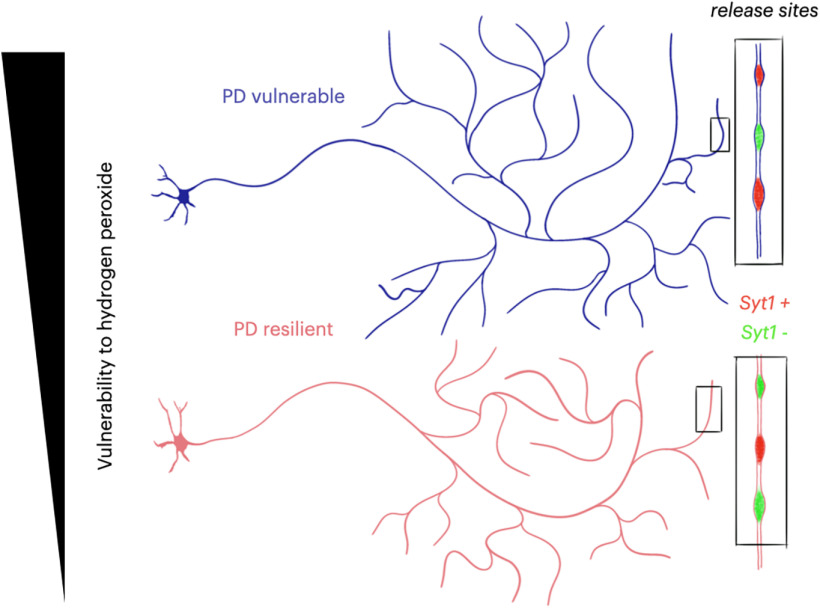

## Significance Statement

Parkinson’s disease (PD) causes the specific degeneration of a small number of neuroanatomically and neurochemically defined neuronal populations. Current hypotheses suggest that these neurons are vulnerable because of their specific physiology and morphology. In this study, using mouse primary neurons, we demonstrate that, when compared with neuronal populations that are suspected to be resilient in PD, neuronal populations that are more vulnerable in PD are more sensitive to cell stress induced by hydrogen peroxide, and that the overt length and complexity of their axonal arborizations is larger. Furthermore, vulnerable neurons show a strikingly elevated proportion of axonal varicosities that are positive for synaptotagmin 1 (Syt-1), suggesting that they contain more active axon terminals.

## Introduction

There are no disease modifying treatments available for people living with Parkinson’s disease (PD). This is clearly related to our limited understanding of why varying PD-related risk factors converge in causing selective dysfunction and degeneration of several neurochemically and anatomically restricted neuronal populations. A better understanding of the origin of selective neuronal vulnerability in PD is therefore critical.

Canonically, PD pathology is described by the presence of Lewy bodies in the brain (for historical review, see Goedert et al., 2013) and by the loss of neuromelanin-containing dopamine (DA) neurons in the substantia nigra pars compacta (SNc). However, the relationship between Lewy pathology and cell loss in PD is unclear and has not unequivocally revealed why certain neurons degenerate, while others do not (Surmeier et al., 2017a; Espay and Lang, 2018). Importantly, the pathology and degeneration in PD is not limited to DA neurons of the SNc, but rather appears to occur in several nuclei, including the locus coeruleus (LC; Huynh et al., 2021) and pedunculopontine nucleus (Hirsch et al., 1987; Tubert et al., 2019). However, more systematic stereological quantifications are clearly required to confirm the nuclei showing frank cell loss and not only Lewy pathology, as well as the sequence of this cell loss (Giguère et al., 2018). A better understanding of what determines the vulnerability of neurons that are affected in PD is essential for progress (Surmeier et al., 2017b).

An increasing amount of work supports the hypothesis that the neurons that are most vulnerable in PD share distinguishing morphologic and physiological characteristics, cell-autonomous factors, that render them selectively vulnerable. Among these, two have been increasingly gaining experimental support. The first is autonomous pacemaking, with broad spikes, and high intracellular calcium oscillations (Surmeier et al., 2017b). The second is being endowed with a very long, and highly branched axonal domain, with orders of magnitude more neurotransmitter-release sites than most other neuron types (Parent and Parent, 2006; Pissadaki and Bolam, 2013; Pacelli et al., 2015; Wong et al., 2019). It is thought that these features converge in leading to elevated bioenergetic demands and an associated high level of chronic oxidative stress (Pissadaki and Bolam, 2013), making the neurons less resilient to mitochondrial dysfunction, proteostatic burden (Lehtonen et al., 2019), or dysfunction in essential cellular systems such as the endolysosomal system (Vidyadhara et al., 2019).

However, two important foundations underlying this working hypothesis require additional investigation. One, is the purported identity of PD-vulnerable neurons, which is still unclear because comparative stereological counting of multiple nuclei in postmortem brains from PD subjects has not been achieved (Giguère et al., 2018; Lunt et al., 2021). And two, work comparing cell-autonomous factors in PD-vulnerable neuronal populations has been mainly limited to a comparison of SNc and VTA neurons (albeit some exceptions; Goldberg et al., 2012; Sanchez–Padilla et al., 2014).

Previous work has shown that cell-autonomous differential vulnerability of SNc and VTA DA neurons is maintained *in vitro*, including a striking correlation between bioenergetic demands, vulnerability to PD-relevant cell stress, and axonal arbor size (Pacelli et al., 2015). In the present study, we use a similar *in vitro* system to examine the characteristics and vulnerability of several PD-relevant neuronal populations, with the objective to evaluate the hypothesis that the outright size, complexity, and extensive number of neurotransmitter release sites is linked to vulnerability. We compared neurons from regions suspected to be vulnerable in PD [DA neurons of the SNc, noradrenergic neurons of the locus coeruleus (LC), serotonin neurons of the raphe nuclei (R), and cholinergic neurons of the dorsal motor nucleus of the vagus (DMV)], to neurons from regions classically hypothesized as more resilient in PD (DA neurons of the VTA, although their vulnerability in PD is controversial; Alberico et al., 2015), cholinergic neurons of the hypoglossal nucleus (XII) and cholinergic interneurons of the dorsal striatum (STR).

We find that globally, neurons previously suspected to be vulnerable in PD are less resilient to cell stress induced by hydrogen peroxide, except for cholinergic neurons of the DMV. In keeping with the hypothesis of a link between axonal domain complexity and vulnerability, we also find that these vulnerable neurons, on average, have longer and more complex axonal domains, with a much higher proportion of varicosities containing the Ca^2+^ sensor synaptotagmin 1 (Syt-1) and that are thus likely active. Together, these findings support the notion of a link between axonal complexity and basal vulnerability of neurons in the context of oxidative stress.

## Materials and Methods

### Animals

Procedures with animals and their care were conducted in accordance with the Guide to care and use of Experimental Animals of the Canadian Council on Animal Care (Canadian Council on Animal Care, 1993). Experimental protocols were approved by the animal ethics committees of the Université de Montréal. Housing was at a constant temperature of 21°C and humidity of 60%, under a fixed 12/12 h light/dark cycle, with food water available *ad libitum*.

### Transgenic animals used

All mice were maintained as heterozygotes.

### Tyrosine hydroxylase (TH)-green fluorescent protein (GFP)

The TH-GFP transgenic mouse line TH-EGFP/21–31, which carries the enhanced GFP gene under control of the TH promoter (Matsushita et al., 2002), was maintained on a C57BL/6J background.

### DAT-Ai9

Dat-Ires-Cre animals (catalog #006660, The Jackson Laboratory; Bäckman et al., 2006) were crossed with Ai9/tdTomato mice (catalog #007905, The Jackson Laboratory; Madisen et al., 2010), allowing conditional expression of the red fluorescent protein tdTomato in DA neurons. Both of these lines are on a C57BL/6J background.

### ChAT-Ai9

ChAT-IRES-Cre (catalog #006410, The Jackson Laboratory; Rossi et al., 2011) were crossed with Ai9/tdTomato mice (catalog #007905, The Jackson Laboratory; Madisen et al., 2010) allowing conditional expression of the red fluorescent protein tdTomato in cholinergic neurons. The ChAT-IRES-Cre mice are on a C57BL/6;129S6 background.

### SERT-Ai9

Slc6a4-Cre (MMRRC stock #031028-UCD; Gong et al., 2007) were crossed with Ai9 (catalog #007905, The Jackson Laboratory; Madisen et al., 2010) mice, allowing conditional expression of the red fluorescent protein tdTomato in serotonin neurons. The SERT-Cre mice were on a C57BL/6J background.

### Primary cell culture

**Figure 1.**
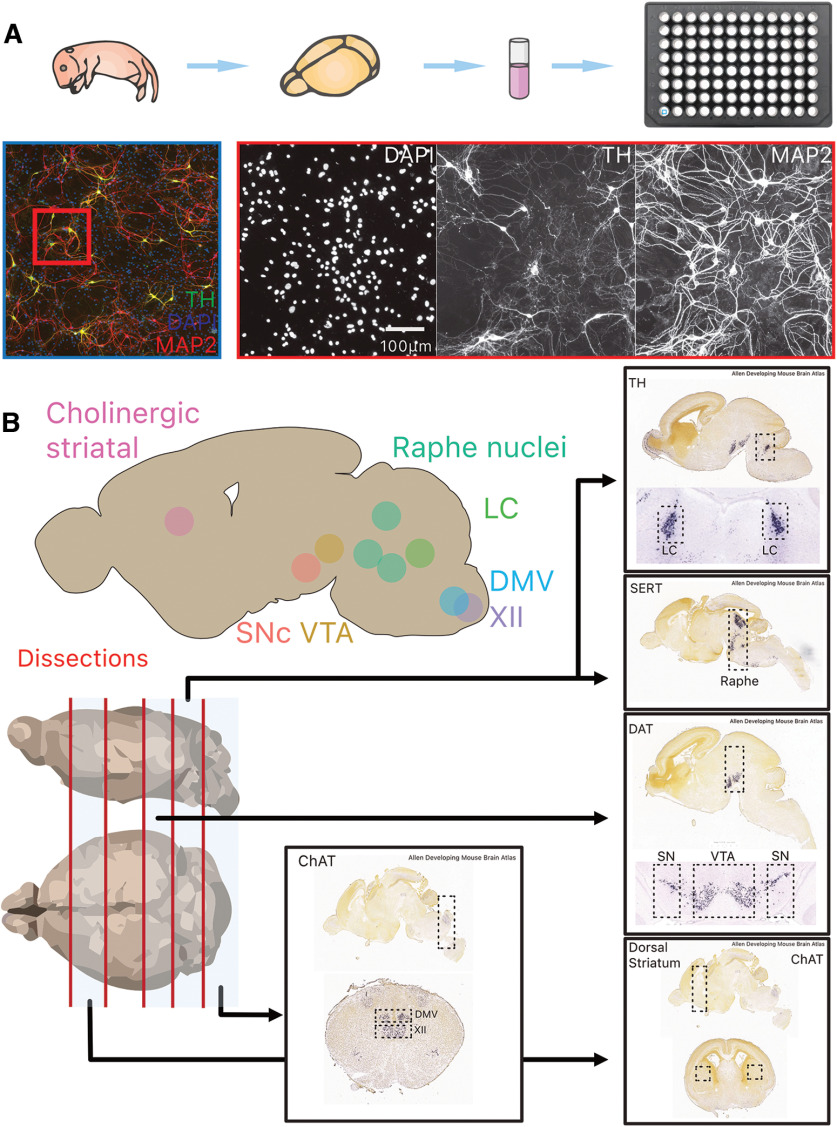
Overview of experimental paradigm, image processing pipeline and neuroanatomical regions and identify of target structures used for primary cultures. ***A***, The brain of postnatal day 0–2 mouse pups were dissected, and target structures isolated before cell dissociation and culture in 96-well plates for 10 DIV. ***B***, Overview of the eight target structures and subsequent dissection strategy in four transgenic mouse lines (TH-GFP, DAT-Ai9, ChAT-Ai9, and SERT-Ai9). Additional images are from the Allen Developing Mouse Brain Atlas (Lein et al., 2007; Allen Brain Atlas, 2008). TH, tyrosine hydroxylase; MAP2, microtubule associated protein 2; DAPI: 4′,6-diamidino-2-phenylindole; LC, locus ceoruleus; DMV, dorsal motor nucleus of the vagus; XII, hypoglossal nucleus; SNc, substantia nigra pars compacta; VTA, ventral tegmental area; SERT, serotonin transporter; DAT, dopamine transporter; ChAT, choline acetyl-transferase.

Primary neuron cultures were prepared from dissections of male and female P0 mice as described previously (Fasano et al., 2008) with slight modification: the manual dissection of brain nuclei containing target neurons inevitably results in uncontrollable differences in the ratio of target cells of interest to other cell types. All culture conditions were identical prior to neuronal seeding, where the only difference was the target region being plated (Fig. 1*A*). Figure 1*B* shows the anatomic location of target structures used in this work. The fluorescence of target structures was used to enable accurate dissection of target nuclei. For all experiments, cells were cultured for 10 d, before either fixation, or live cell imaging. All experiments were performed on at least three (three to six) independent cultures. For analysis of varicosities, cultures were prepared as previously described, and seeded onto 15-mm cell adhesion-treated glass coverslips (65 μl of 100,000 cells/ml; Fasano et al., 2008).

### 96-Well plates

For cell stress assays and neurite tracing, primary cultures were prepared as described above, but seeded into 96-well plates (μ-Plate 96 Well Black, ibiTreat: #1.5 polymer coverslip, tissue culture treated, sterilized; catalog #89626). Since dissections of tissue surrounding targeted neurochemically and anatomically defined nuclei can vary, we pooled cells from three to five postnatal day 0–2 pups, per multiwell plate. This enables a reduction in, for example, total DA SNc neurons variability, culture-to-culture, even where total number of cells (neurons and glia) seeded varied. This resulted in ∼5000 to 10000 cells, in total, being seeded per well. For cell stress assays, hydrogen peroxide [30% (W/W) solution, Sigma H-4381] was added at 0, 100, 150, and 200 μm, at 10 d *in vitro* (DIV), and cells were fixed at 11 DIV.

### Live cell imaging

For live cell imaging, cultures were prepared as above, but seeded (200 μl of 100 000 cells/ml) into 35-mm imaging compatible glass-bottomed Petri dishes [μ-Dish 35 mm, high ibiTreat: 35 mm, high wall (2-ml volume), #1.5 polymer coverslip, tissue culture treated, sterilized, catalog #81156].

### Immunocytochemistry

Cultures were fixed with 4% paraformaldehyde (PFA; in PBS, pH 7.4) at 10 DIV, permeabilized with 0.1% Triton X-100 during 20 min, and nonspecific binding sites were blocked with 10% bovine serum albumin for 10 min. Primary antibodies were incubated overnight at room temperature: anti-TH (1:2000, AB152, Cedarlane), anti-MAP2 (1:2000, MAB3418, Millipore Sigma), anti-RFP (1:1000, 600-401-379, Cedarlane), anti-Syt-1 (1:200, 105102, Synaptic Systems). These were subsequently detected using Alexa Fluor-488-conjugated, Alexa Fluor-546-conjugated, Alexa Fluor-568-conjugated, or Alexa Fluor-647-conjugated secondary antibodies (incubated at room temperature at 1:1000 for 1 h, Invitrogen).

### Mitochondrial targeting of the reactive oxygen species (ROS) sensor, roGFP

For all primary neuron cultures in a cre background, we expressed a MTS (mitochondrially targeted sequence) form of roGFP by adding 1 μl of AAV9-CMV-DIO-MTS-roGFP-WPRE-bGHpA (∼2–3 × 10^13^ vector genome ml^−1^ titers) to cultures at 1 DIV. For cultures targeting MTS-roGFP to LC neurons, in C57 primary neurons, 1 μl AAV9-TH-MTSroGFP (∼2–3 × 10^13^ vector genome ml^−1^ titers) was used. These tools (Sanchez–Padilla et al., 2014) were kind gifts from the laboratory of D. James Surmeier (Department of Physiology, Feinberg School of Medicine, Northwestern University, Chicago, IL).

### Imaging and data analysis

#### Image acquisition

Confocal imaging was conducted on an Olympus Fluoview FV1000 point-scanning confocal microscope (Olympus). Images were scanned sequentially to prevent nonspecific bleed-through signal using 488-, 546-, and 647-nm laser excitation and a 60× (NA 1:42) oil immersion objective. All other imaging was acquired on a Nikon Eclipse Ti2-E inverted microscope, using either a CFI Plan Apo λ 20× objective (for cell counting and neurite tracing), or a CFI Plan Apo λ 60× oil immersion objective (for live cell imaging).

#### Image processing and analysis, data analysis, and statistics

Exploratory image visualization and analysis was done using Napari (Sofroniew et al., 2021). Subsequently, unmodified images were all processed using ImageJ (Schneider et al., 2012) and custom analysis scripts written in ImageJ Macro language (https://github.com/samuelorion/burke-trudeau-2022).

#### Raw data availability

Because of the considerable volume of imaging data (>5 TBs) generated in the present study, sharing of the primary data on an open data-storage solutions was not possible. However, these data are available on request. Derived data are available at https://github.com/samuelorion/burke-trudeau-2022.

### Image analysis

#### Counts and neurite tracing

To conduct unbiased and high-throughput quantifications of neuron numbers, we developed our own methods to count projecting neurons. These analysis scripts can be found in the associated GitHub repository. Briefly, images were processed for segmentation to identity cell bodies. Segmentations were then compared with raw images and validated for accuracy, where we achieved ∼90% accuracy across neuron types. A slightly modified version (https://github.com/samuelorion/burke-trudeau-2022) of the above system was adapted to achieve similar performance for tracing and quantification of neurites.

#### ROS quantification

Imaging experiments were performed at room temperature (20–22°C) because previous studies showed that probe oxidation was nearly complete at physiological temperatures (Sanchez–Padilla et al., 2014). Regions of interest (ROIs) were generated using an automatic segmentation approach (https://github.com/samuelorion/burke-trudeau-2022), where GFP-positive puncta were segmented and used to measure fluorescence intensity. Recordings where a drifting baseline of >10% was detected, because of photobleaching or photo-oxidation of roGFP, were not included. The maximum and minimum fluorescence of mito-roGFP was determined according to a previously described procedure (Guzman et al., 2010), by application of 2 mm dithiothreitol (DTT; to reduce the mitochondria fully), and then 100 μm aldrithiol (ALD; to oxidize the mitochondria fully). ROIs that did not responds to DTT and ALD were not included. The relative oxidation level was then calculated as 1 − [(F − FALD)/(FDTT − FALD)], where *F* represents fluorescence intensity at baseline, FDTT, F in the presence of DTT, and FALD, F in the presence of ALD. Image alignment was done using the ImageJ plugin StackReg (Thévenaz et al., 1998). For quantifications of intermitochondrial distances, the distance between roGFP positive puncta was manually measured using Napari. Mitochondria in the somatodendritic (STD) compartment were not quantified because of the very high density of mitochondria, making this quantification unfeasible.

### Varicosities

Confocal images were processed (https://github.com/samuelorion/burke-trudeau-2022) and varicosities were segmented. Varicosities were defined as enlargements along thin neurites with a measured width between 0.2 and 1 μm, and a length of 0.3–0.5 μm. Segmentations were then mapped onto images of Syt-1 immunofluorescence signal and fluorescence intensity was quantified. To determine the status of Syt-1 positivity (Figs. 7*A*, 8*B*), we estimated the intensity of Syt-1 signal in segmentations that were excluded and used this value as a cut off for Syt-1-positive status.

### Statistics

Given previous work in our group quantifying neuron numbers on coverslips, we conducted an a priori power analysis to detect an effect size of 25%, with a power of 80%, and an α = 0.05, concluding our requirement for n to be 22 for survival assays. We therefore aimed for this. However, for some wells, images were excluded because of contamination. For all other experiments, we aimed to have an n of at least 12. For statistical analyses and data visualization we used R (R Core Team, 2017), and the subsequent packages for all statistical analyses, and data visualization (RStudio Team, 2020; Ho and Tumkaya, 2020; Müller, 2020; Ogle et al., 2021; Pedersen, 2021; Slowikowski, 2021; Wickham, 2021; Wilke, 2021]. For each statistical analysis, we evaluated whether the data were parametric or nonparametric, and subsequently conducted appropriate analyses, including appropriate *post hoc* multiple comparisons: described in the available supplementary .Rmd file (https://github.com/samuelorion/burke-trudeau-2022). In figures, we include an asterisk to indicate that, for an α = 0.05, there is a significant difference compared with the value for the SNc (our reference neuronal population). All other *post hoc* comparisons can be found in the extended data tables. All figures include box and whiskers plots, in the style of Tukey, where we indicate the median value, and the lower and upper hinges correspond to the first and third quartiles (the 25th and 75th percentiles), and the whiskers extend to the largest and smallest value (no further than 1.5 times the interquartile range). Furthermore, where appropriate, we include individual data points. Following null hypothesis testing, we also performed estimation based on confidence intervals using the R package *dabestr* (Ho et al., 2019). For all experiments, we conducted shared control Cumming plots, compared with the SNc. These Cumming plots have been placed below plots, below null hypothesis testing. Furthermore, analyses were conducted comparing neurons considered vulnerable in PD to neurons considered more resilient in PD, where Gardner–Altman two-group estimation plots are plotted next to main box and whisker plots.

## Results

We used a mouse primary culture system in which neurons from multiple brain PD-relevant regions were grown on supporting astrocytes (Fig. 1*B*). These were subsequently neurochemically defined, identifying them either by immunocytochemistry for their neurochemical identity, or the presence of the fluorescent reporter protein TdTomato, expressed in a cre-dependent manner. For this study, we chose four nuclei that are considered vulnerable in PD, including DA neurons of the SNc, noradrenergic neurons of the LC, serotonin neurons of the raphe nuclei, and cholinergic (ChAT+) neurons of the dorsal motor nucleus of the vagus (DMV). We compared these to neuronal populations considered more resilient in PD, including DA neurons of the VTA, cholinergic neurons of the hypoglossal nucleus (XII), and cholinergic interneurons of the dorsal striatum (STR).

### Neuronal populations considered vulnerable in PD are less resilient to cell stress induced by hydrogen peroxide

**Figure 2.**
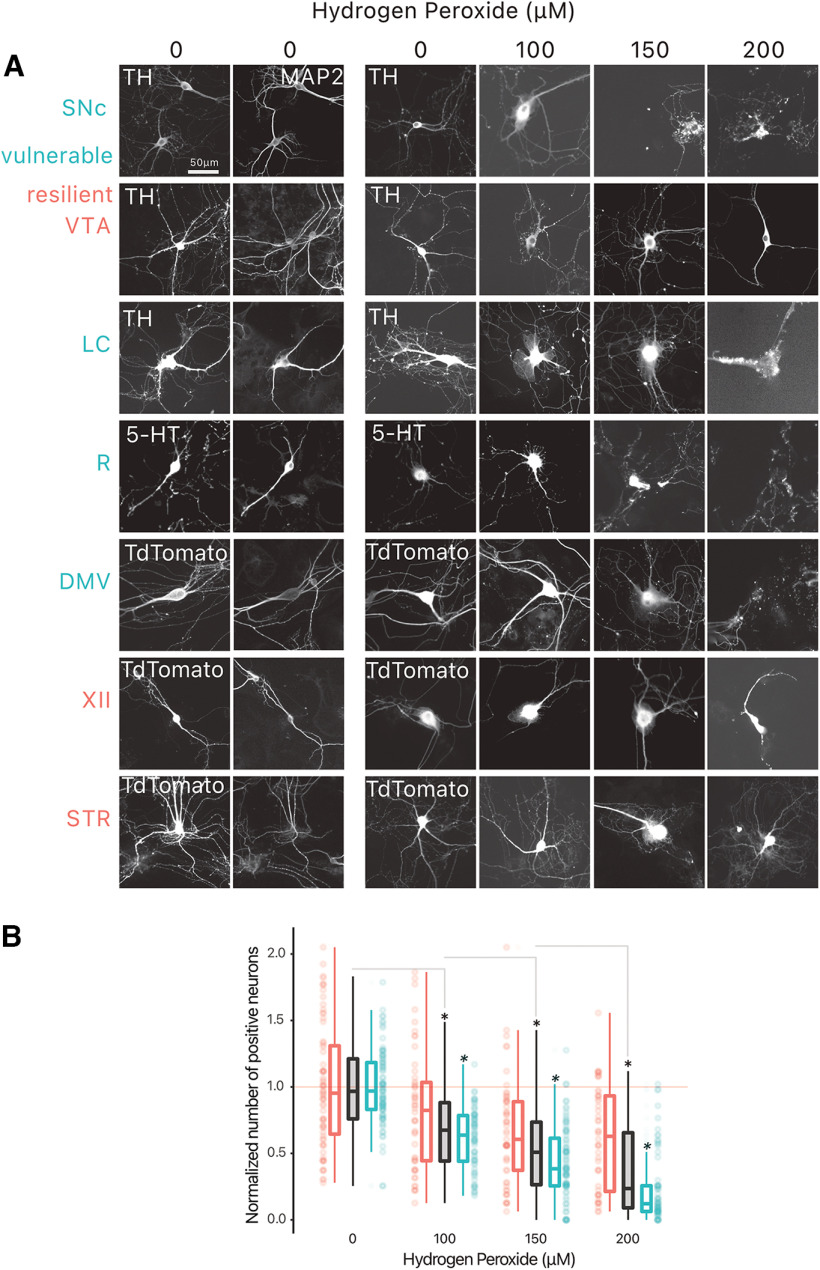
Vulnerable neurons are more vulnerable to hydrogen peroxide than resilient neurons. Neurons were treated with hydrogen peroxide at 10 DIV, and fixed at 11 DIV. ***A***, Example photomicrographs of all positive-identified neuron types across the vulnerable, and resilient target structures. ***B***, Normalized number of positive neurons across hydrogen peroxide concentrations. Box and whiskers plots, in the style of Tukey, where the median value is indicated, and the lower and upper hinges correspond to the first and third quartiles. * = one-way ANOVA, Tukey’s HSD test, *p* < 0.05; * = pairwise *t* test, vulnerable versus resilient, *p* < 0.05. Detailed statistical tests and multiple comparisons can be found in Extended Data Table 2-1. TH, tyrosine hydroxylase; MAP2, microtubule associated protein 2; LC, locus ceoruleus; DMV, dorsal motor nucleus of the vagus; XII, hypoglossal nucleus; SNc, substantia nigra pars compacta; VTA, ventral tegmental area; R, raphé; STR, striatum.

Previous work has shown that in line with *in vivo* observations, DA neurons of the SNc *in vitro* are more vulnerable than DA neurons of the VTA to cell stress induced by several PD-relevant stressors (Pacelli et al., 2015; Lieberman et al., 2017). Here, we extended such a comparative analysis of neuronal vulnerability in a much larger set of neuronal populations with distinct neurochemical identities. Considering the well-established contribution of oxidative stress to PD pathophysiology, we compared the survival of neurons exposed to three concentrations of hydrogen peroxide (100, 150 and 200 μm), a cell stressor that is expected to act on all types of neurons (Fig. 2). A quantification of neurochemically-defined neurons in these cultures revealed that, considered as a group, neurons from “vulnerable” nuclei (SNc, LC, R, DMV) were more sensitive to hydrogen peroxide compared with neurons from “resilient” nuclei (VTA, XII, STR; Fig. 2*B*; Extended Data Table 2-1).

10.1523/ENEURO.0139-22.2022.tab2-1 Extended Data Table 2-1 Statistical reporting for Figure 2*B*. Download Table 2-1, DOCX file.

**Figure 3.**
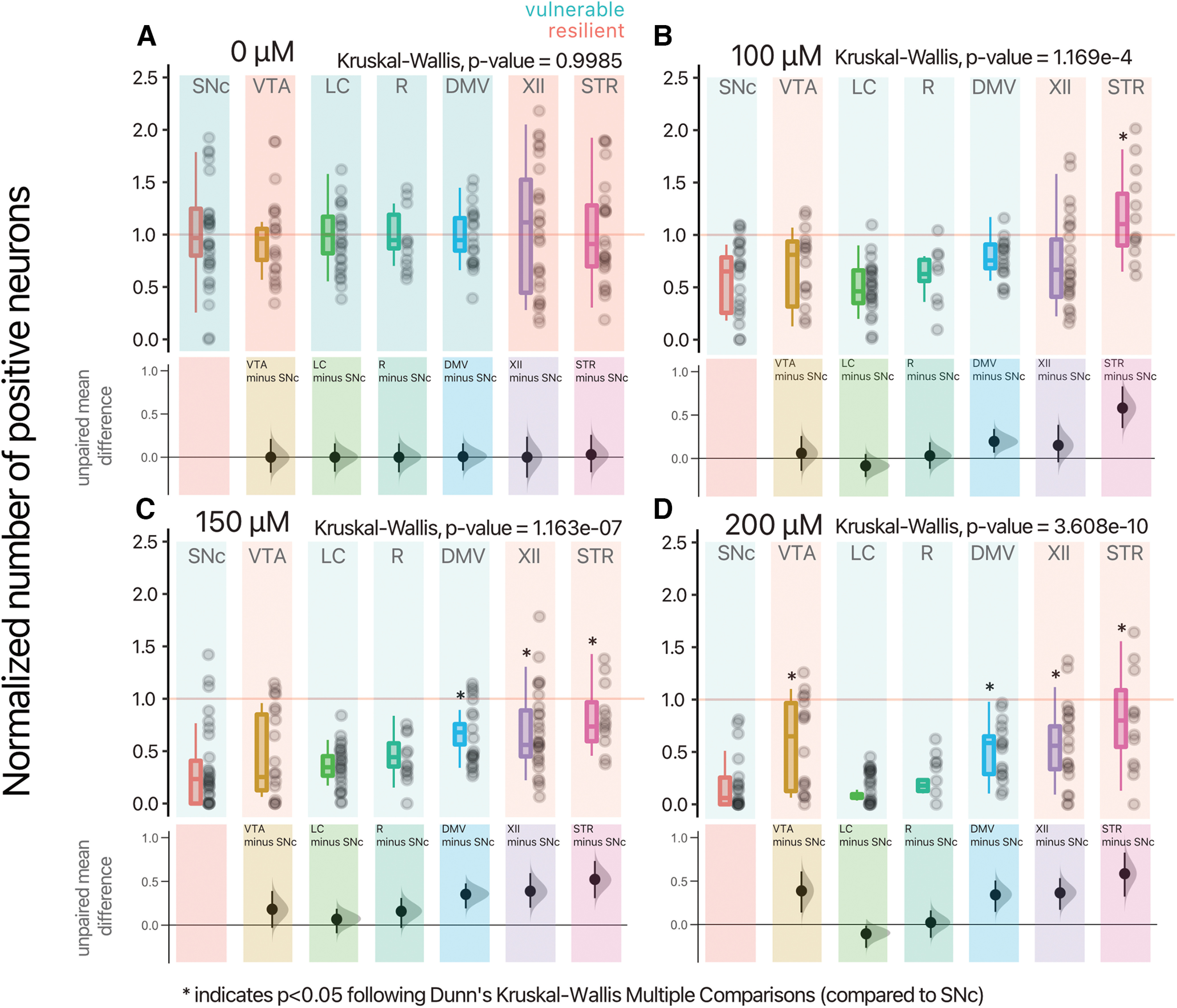
Differential vulnerability between neurons to hydrogen peroxide. ***A–D***, Normalized number of positive-neurons, for each concentration of hydrogen peroxide, and neuronal population. Box and whiskers plots, in the style of Tukey, where the median value is indicated, and the lower and upper hinges correspond to the first and third quartiles, Kruskal–Wallis multiple comparison, *p*-values adjusted with the Bonferroni method, *p < 0.05. Shared control estimation plot: mean difference for comparisons against the shared control, SNc, using Data Analysis with Bootstrap Estimation, with 5000 bootstrap resamples. All confidence intervals are bias-corrected and accelerated. Detailed statistical tests and multiple comparisons can be found in Extended Data Table 3-1. LC, locus ceoruleus; DMV, dorsal motor nucleus of the vagus; XII, hypoglossal nucleus; SNc, substantia nigra pars compacta; VTA, ventral tegmental area; R, raphé; STR, striatum.

A closer examination of the relative vulnerability of each neuronal population across the increasing doses of hydrogen peroxide (Fig. 3) revealed significant effects of hydrogen peroxide at doses of 100, 150, and 200 μm (Extended Data Table 3-1). Notably, at 150 μm hydrogen peroxide, cholinergic neurons of the DMV, hypoglossal nucleus and striatum showed less neuronal loss compared with SNc DA neurons, ChAT+ neurons of the DMV [unpaired mean difference of DMV (*n* = 16) minus SNc (*n* = 20) 0.197 [95CI 0.0662; 0.34]], ChAT+ neurons of the XII [unpaired mean difference of XII (*n* = 23) minus SNc (*n* = 20) 0.152 [95CI −0.0443; 0.388]], and ChAT+ neurons of the STR [unpaired mean difference of STR (*n* = 12) minus SNc (*n* = 20) 0.581 [95CI 0.35; 0.831]]. In comparison, LC and Raphe neurons showed cell loss comparable to SNc DA neurons. VTA DA neurons also showed a tendency for reduced cell loss compared with SNc DA neurons, although this did not reach significance (Fig. 3). This differential vulnerability to hydrogen peroxide persisted at 200 μm, with VTA DA neurons and all cholinergic groups showing reduced vulnerability (Fig. 3). Except for the DMV, hypothesized to be vulnerable in PD, our findings are in keeping with the hypothesis that in addition to DA neurons of the SNc, noradrenergic LC neurons and serotonergic Raphe neurons show elevated intrinsic vulnerability.

10.1523/ENEURO.0139-22.2022.tab3-1 Extended Data Table 3-1 Statistical reporting for Figure 3*A–D*. Download Table 3-1, DOCX file.

### No overt difference in mitochondrial ROS production is observed between neurons

**Figure 4.**
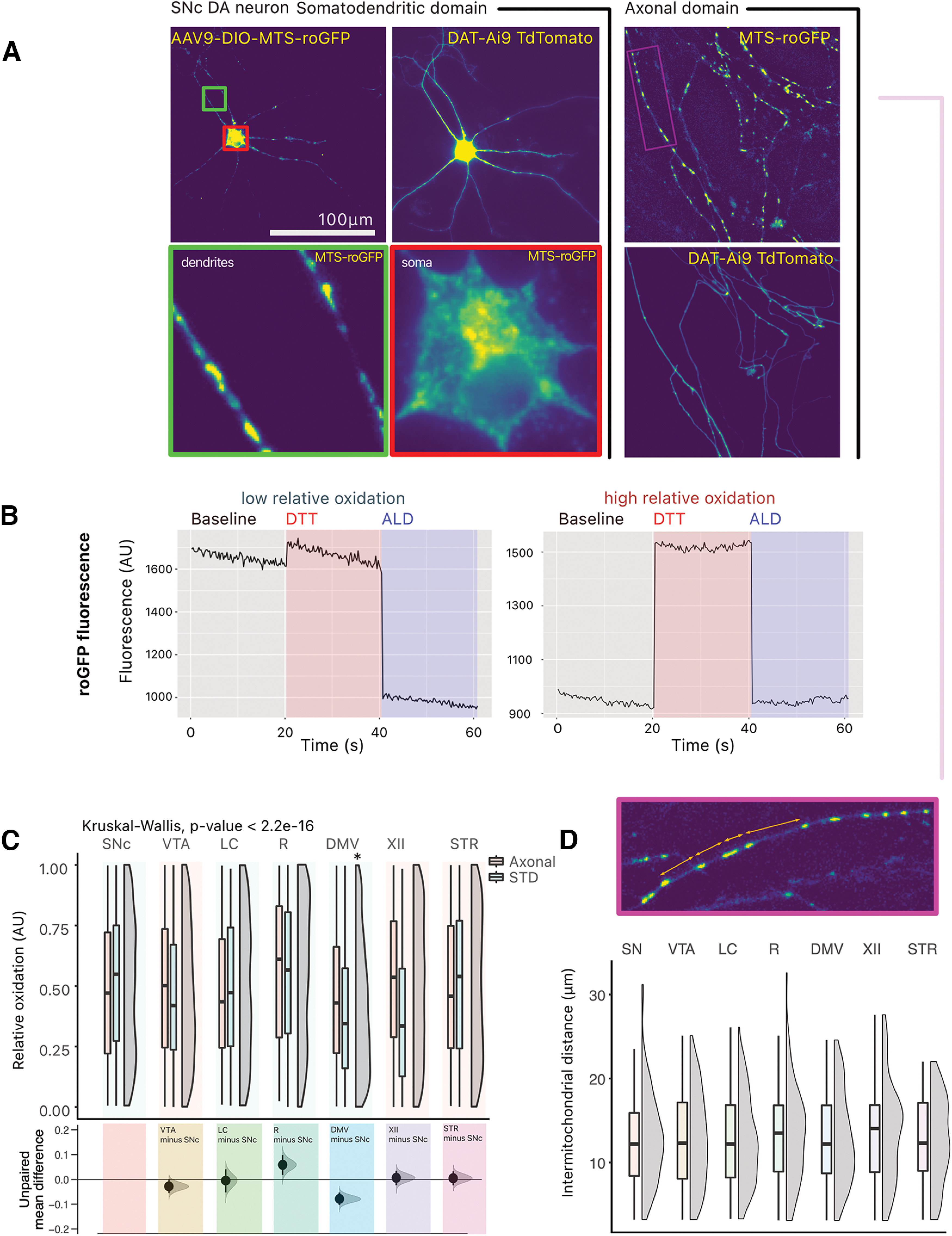
No overt difference in ROS production is observed between neurons. ***A***, The redox sensitive GFP, roGFP, was expressed in a cre-dependent manner in target neurons. Photomicrographs of roGFP in a TdTomato-positive SNc DA neuron, in the somatodendritic (STD) compartment and in the axonal compartment. ***B***, Example traces of GFP fluorescence arbitrary units (AU) in responsive ROIs, in an SNc DA neuron. Two traces are shown: for a GFP-positive puncta showing low relative oxidation status, and for a GFP-positive puncta showing high relative oxidation status. ***C***, Quantification of the relative oxidative state of mitochondria across neuron types. Box and whiskers plots, in the style of Tukey, where the median value is indicated, and the lower and upper hinges correspond to the first and third quartiles, Kruskal–Wallis multiple comparison, *p*-values adjusted with the Bonferroni method, *p < 0.05. Shared control estimation plot: mean difference for comparisons against the shared control, SNc, using Data Analysis with Bootstrap Estimation, with 5000 bootstrap resamples. All confidence intervals are bias-corrected and accelerated. ***D***, Quantification of intermitochondrial distance in the axonal domain (measured from the center of each GFP positive puncta). Box and whiskers plots, in the style of Tukey, where the median value is indicated, and the lower and upper hinges correspond to the first and third quartiles, Kruskal–Wallis *p* > 0.05. Density plots show distribution of individual measurements. Detailed statistical tests and multiple comparisons can be found in Extended Data Tables 4-1 and 4-2. Extended Data Figure 4-1 also provides micrographs showing the expression of MTSroGFP in locus coeruleus neurons and comparative data on the relative oxidation in the somatodendritic and axon domain of the different neuron types, as well as the normalized number of DAPI-positive nuclei in the neuron survival experiments performed with hydrogen peroxide. LC, locus ceoruleus; DMV, dorsal motor nucleus of the vagus; XII, hypoglossal nucleus; SNc, substantia nigra pars compacta; VTA, ventral tegmental area; R, raphé; STR, striatum.

The origin of the elevated vulnerability of SNc and other vulnerable neurons in PD has been previously hypothesized to result at least in part from their particularly elevated bioenergetic demands, leading to higher rates of mitochondrial oxidative phosphorylation and chronically elevated levels of oxidative stress (Bolam and Pissadaki, 2012; Pacelli et al., 2015). We therefore examined basal mitochondrially-derived ROS production using the ROS sensitive GFP probe, mito-roGFP. The construct was expressed in neurons using a Cre recombinase-dependent AAV vector or a TH promotor (for LC neurons; Fig. 4*A*). The selective expression of mito-roGFP was validated by confirming that expression was limited to tdTomato-expressing neurons or to neurons positive for TH (for LC neurons, see Extended Data Fig. 4-1*A*). Experiments were conducted by live time-lapse imaging of baseline mito-roGFP fluorescence, followed by a determination of the dynamic range of the reporter by measuring the fluorescence increase induced by the reducing agent DTT and the fluorescence decrease induced by the oxidant molecule aldrithiol (ALD; Fig. 4*B*). The signal was quantified both in the neurons’ STD domain and in the neurons’ axonal fields (Fig. 4*A*). Among the neurons examined, a broad range of basal oxidant levels were identified, with some mitochondria within neurons showing low basl oxidation and others showing high basal oxidation (Fig. 4*C*). These experiments revealed that, contrary to expectation, only ChAT+ DMV neurons had significantly reduced relative oxidation, when compared with the DA SNc neurons (Fig. 4*C*; Extended Data Table 4-1). Furthermore, intermitochondrial distances within the axonal domain were comparable across neuron types, with a mean of ∼13 μm (SD of 5.4; Fig. 4*D*; Extended Data Table 4-2).

10.1523/ENEURO.0139-22.2022.f4-1 Extended Data Figure 4-1 ***A***, Expression of MTSroGFP in locus coeruleus neurons. Photomicrographs of a LC noradrenergic neuron infected with AAV9-TH-MTSroGFP. The neuron is identified by the presence of TH (red). MTSroGFP is shown in green. Nuclei, stained with DAPI, are shown in blue. ***B***, Comparing relative oxidation in neuron types and in somatodendritic domain and axons shows only very small differences. Compared with Welch two-sample *t* test. ***C***, Normalized number of nuclei (DAPI-positive) across hydrogen peroxide concentrations. Box and whiskers plots, in the style of Tukey, where the median value is indicated, and the lower and upper hinges correspond to the first and third quartiles. * = one-way ANOVA, Tukey’s HSD test, *p* < 0.05; * = pairwise *t* test, vulnerable versus resilient, *p* < 0.05. Download Figure 4-1, TIF file.

10.1523/ENEURO.0139-22.2022.tab4-1 Extended Data Table 4-1 Statistical reporting for Figure 4*C*. Download Table 4-1, DOCX file.

10.1523/ENEURO.0139-22.2022.tab4-2 Extended Data Table 4-2 Statistical reporting for Figure 4*D*. Download Table 4-2, DOCX file.

### Neuronal populations considered vulnerable in PD have large axonal domains, which are more complex than neurons considered as more resilient

**Figure 5.**
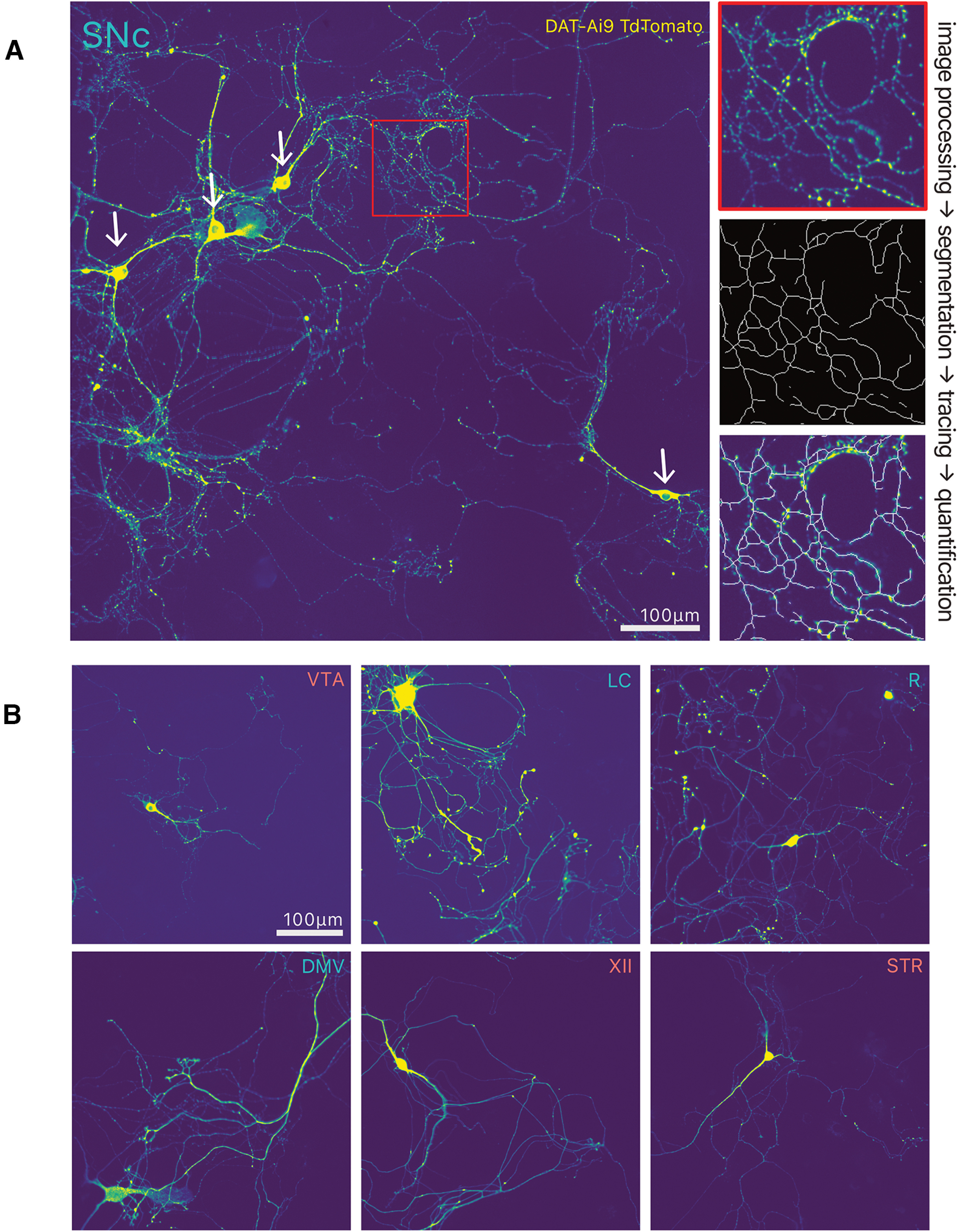
Overview of neurite tracing for quantification. ***A***, Photomicrograph of SNc DA neurons, and overview of neurite quantification method. ***B***, Photomicrographs illustrating the different types of neurons examined, with their neurochemical marker immunocytochemistry. LC, locus ceoruleus; DMV, dorsal motor nucleus of the vagus; XII, hypoglossal nucleus; SNc, substantia nigra pars compacta; VTA, ventral tegmental area; R, raphé; STR, striatum.

**Figure 6.**
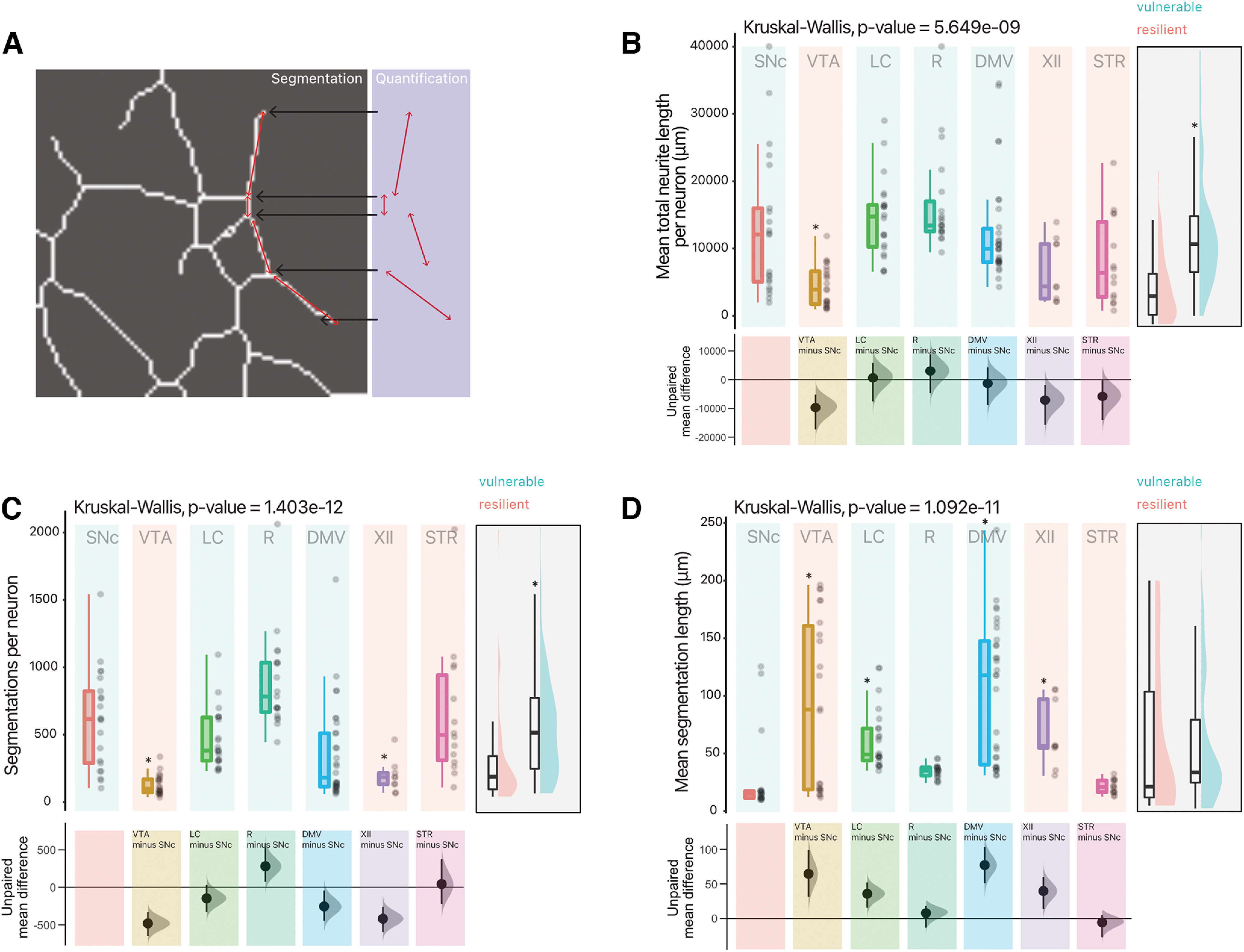
Vulnerable neurons have large axonal domains, which are globally more complex than resilient neurons. ***A***, Overview of quantification method for neurite segmentations. ***B***, Quantification of mean neurite length (total neurite length, per well, divided by number of neurons). Box and whiskers plots, in the style of Tukey, where the median value is indicated, and the lower and upper hinges correspond to the first and third quartiles, Kruskal–Wallis multiple comparison, *p*-values adjusted with the Bonferroni method, *p < 0.05. Shared control estimation plot: mean difference for comparisons against the shared control, SNc, using Data Analysis with Bootstrap Estimation, with 5000 bootstrap resamples. All confidence intervals are bias-corrected and accelerated. ***C***, Quantification of mean number of segmentations (sections) of neurites segmented per neuron. Box and whiskers plots, in the style of Tukey, where the median value is indicated, and the lower and upper hinges correspond to the first and third quartiles, Kruskal–Wallis multiple comparison, *p*-values adjusted with the Bonferroni method, *p < 0.05. Shared control estimation plot: mean difference for comparisons against the shared control, SNc, using Data Analysis with Bootstrap Estimation, with 5000 bootstrap resamples. All confidence intervals are bias-corrected and accelerated. ***D***, Quantification of mean length of segmentations (sections) of neurites segmented per neuron. Box and whiskers plots, in the style of Tukey, where the median value is indicated, and the lower and upper hinges correspond to the first and third quartiles, Kruskal–Wallis multiple comparison, *p*-values adjusted with the Bonferroni method, *p < 0.05. Shared control estimation plot: mean difference for comparisons against the shared control, SNc, using Data Analysis with Bootstrap Estimation, with 5000 bootstrap resamples. All confidence intervals are bias-corrected and accelerated. ***B*** to ***C*** have a supplementary plot of all data grouped as Vulnerable and Resilient, where an independent two-group Mann–Whitney *U* test was performed, and an asterisk denotes *p* < 0.05. Precise values can be found in the supplementary tables alongside unpaired Gardner–Altman two group estimation plots. Detailed statistical tests and multiple comparisons can be found in Extended Data Table 6-1. LC, locus ceoruleus; DMV, dorsal motor nucleus of the vagus; XII, hypoglossal nucleus; SNc, substantia nigra pars compacta; VTA, ventral tegmental area; R, raphé; STR, striatum.

Previous work has shown that murine DA SNc neurons have larger and more complex axonal arbors compared with VTA DA neurons, both *in vitro* (Pacelli et al., 2015) and *in vivo* (Giguère et al., 2019). This observation is in line with the hypothesis that the total length and complexity of the axonal domain is a cell-autonomous feature that contributes to rendering these cells most vulnerable because of its associated bioenergetic burden. Here, given our working hypothesis, we examined the morphology of the axonal domain of each neuronal population. Given the number of neuron subtypes evaluated in this study, we developed simple and robust methods to quantify the axonal domain of these projecting neurons, all quantifications normalized to the number of neurons within each well (Figs. 5*A*,*B*, 6*A*). Given that the majority of neurites detected in these neurons were MAP-2 negative, and thus not dendrites, we considered the neurons’ somatodendritic domain as being negligible in size compared with the axonal domain (Fig. 5*A*) and therefore quantified neurite length as a proxy for axonal length. We find that there is a significant difference in mean total neurite length when comparing vulnerable to resilient neurons (Fig. 6*B*; Extended Data Table 6-1). Surprisingly, only VTA DA neurons were significantly different compared with SNc DA neurons in terms of mean neurite length per neuron [unpaired mean difference of VTA (*n* = 22) minus SNc (*n* = 21) −9450 [95CI −16,900; −5080]]. However, using data analysis with bootstrap estimation suggests that both hypoglossal and striatal cholinergic neurons also have, on average, shorter axonal arborizations [unpaired mean difference of XII (*n* = 19) minus SNc (*n* = 21) −6940 [95CI −15,300; −1850], unpaired mean difference of STR (*n* = 14) minus SNc (*n* = 21) −5660 [95CI −13,700; −190]]. We also estimated axonal arbor complexity by measuring the total number of segmentations and average length of segmentations (Fig. 6*C*,*D*). Vulnerable neurons had far more segmentations per neuron compared with resilient neurons. We find that, compared with SNc DA neurons, VTA DA neurons and hypoglossal neurons have substantially fewer segmentations per neuron (Fig. 6*C*; Extended Data Table 6-1). We finally estimated the average axon branch length, and no overt difference were observed between vulnerable and resilient neurons. However, VTA DA neurons, LC noradrenergic neurons, DMV cholinergic neurons and hypoglossal cholinergic neurons had longer segmented neurite length compared with SNc DA neurons [Fig. 6*D*; Extended Data Table 6-1; unpaired mean difference of VTA (*n* = 22) minus SNc (*n* = 21) 64.6 [95CI 31.4; 99.1], unpaired mean difference of DMV (*n* = 30) minus SNc (*n* = 21) 77.3 [95CI 50.9; 104]]. Together, these results suggest that although inconsistencies were observed, globally, mean total neurite length is smaller in neuronal populations suspected to be more resilient in PD and the total number of neurites is also smaller in these neurons.

### Neuronal populations considered vulnerable in PD have a higher proportion of varicosities that are positive for Syt-1

**Figure 7.**
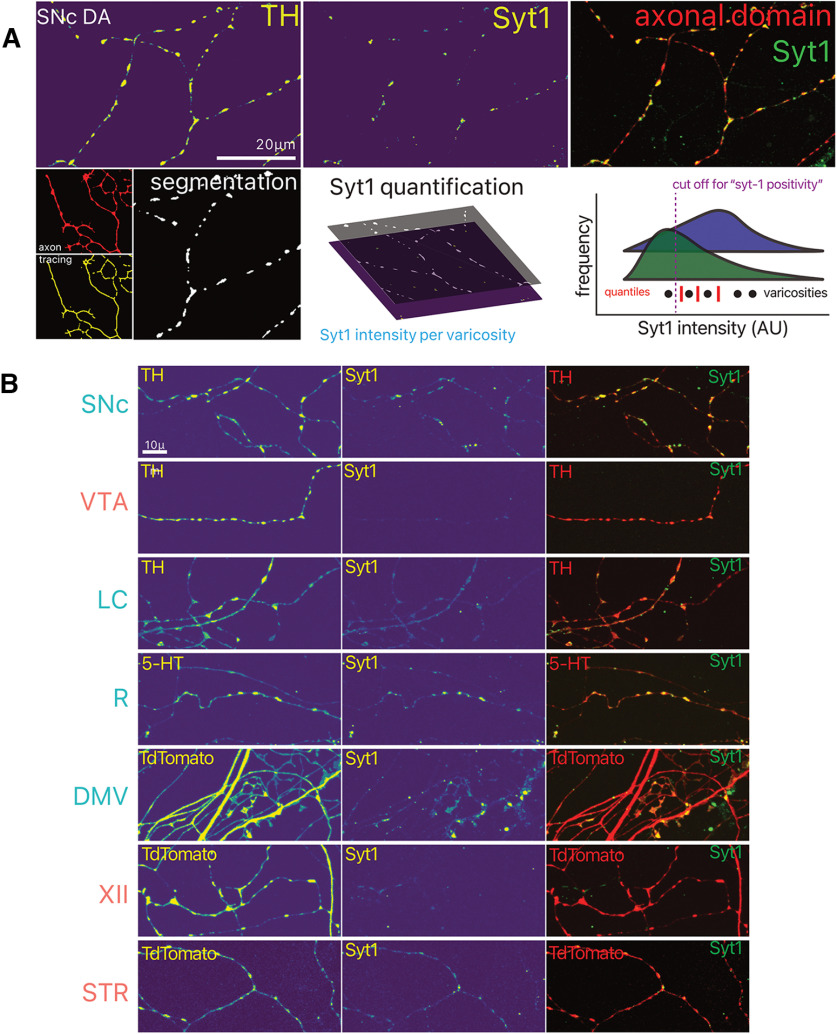
Identification of potential functional axonal varicosities by Syt-1 immunocytochemistry. ***A***, Overview of the image analysis strategy used for quantification of Syt-1 positivity of varicosities, and distribution of Syt-1 intensity within varicosities (bottom right).Arbitrary units (AU) ***B***, Photomicrographs of axonal fields of neurons, with their neurochemical marker and Syt-1 immunocytochemistry. LC, locus ceoruleus; DMV, dorsal motor nucleus of the vagus; XII, hypoglossal nucleus; SNc, substantia nigra pars compacta; VTA, ventral tegmental area; R, raphé; STR, striatum.

**Figure 8.**
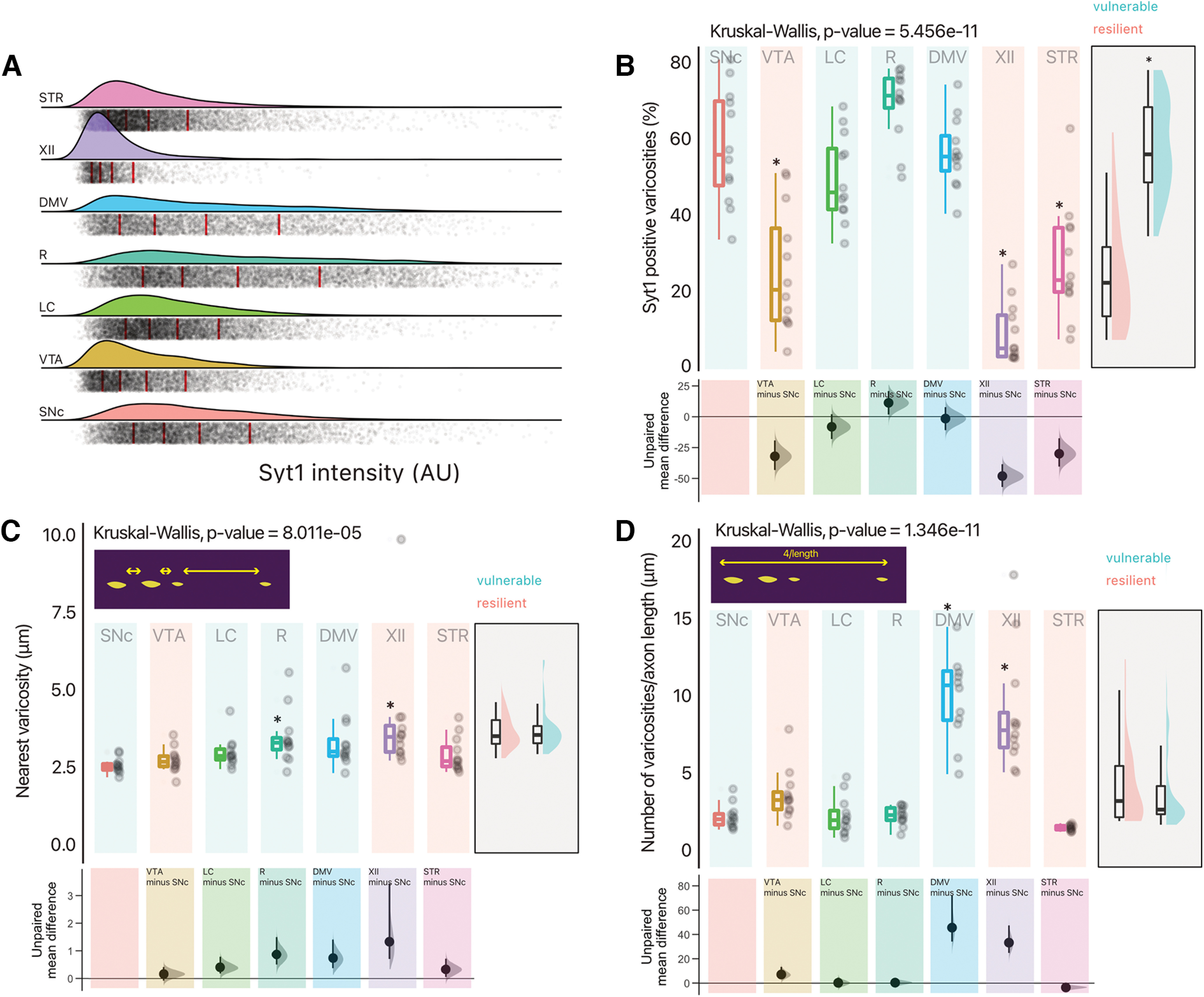
Vulnerable neurons have a higher proportion of varicosities that are positive for Syt-1. ***A***, Density plot of Syt-1 intensity [arbitrary units (AU), fluorescence] in all segmented varicosities included for analysis (red line indicate quintiles). ***B***, Quantification of the proportion of varicosities that are positive for Syt-1. Box and whiskers plots, in the style of Tukey, where the median value is indicated, and the lower and upper hinges correspond to the first and third quartiles, Kruskal–Wallis multiple comparison, *p*-values adjusted with the Bonferroni method, *p < 0.05. Shared control estimation plot: mean difference for comparisons against the shared control, SNc, using Data Analysis with Bootstrap Estimation, with 5000 bootstrap resamples. All confidence intervals are bias-corrected and accelerated. ***C***, Quantification of intervaricose distance (nearest neighbor analysis of segmentations). Box and whiskers plots, in the style of Tukey, where the median value is indicated, and the lower and upper hinges correspond to the first and third quartiles, Kruskal–Wallis multiple comparison, *p*-values adjusted with the Bonferroni method, *p < 0.05. Shared control estimation plot: mean difference for comparisons against the shared control, SNc, using Data Analysis with Bootstrap Estimation, with 5000 bootstrap resamples. All confidence intervals are bias-corrected and accelerated. ***D***, Mean number of varicosities per unit length of axonal domain. Box and whiskers plots, in the style of Tukey, where the median value is indicated, and the lower and upper hinges correspond to the first and third quartiles, Kruskal–Wallis multiple comparison, *p*-values adjusted with the Bonferroni method, *p < 0.05. Shared control estimation plot: mean difference for comparisons against the shared control, SNc, using Data Analysis with Bootstrap Estimation, with 5000 bootstrap resamples. All confidence intervals are bias-corrected and accelerated. ***B*** to ***C*** have a supplementary plot of all data grouped as vulnerable and resilient, where an independent two-group Mann–Whitney *U* test was performed, and an asterisk denotes *p* < 0.05. Precise values can be found in the supplementary tables alongside unpaired Gardner–Altman two group estimation plots. Detailed statistical tests and multiple comparisons can be found in Extended Data Table 8-1. LC, locus ceoruleus; DMV, dorsal motor nucleus of the vagus; XII, hypoglossal nucleus; SNc, substantia nigra pars compacta; VTA, ventral tegmental area; R, raphé; STR, striatum.

Neurotransmitter release sites are known to represent sites of high energy consumption in neurons (Pulido and Ryan, 2021). As such, we hypothesize that their density could represent a defining characteristic of vulnerable neurons in PD. We therefore estimated the density of potential neurotransmitter release sites (varicosities) along the axonal domains of these projecting neurons (Fig. 7*A*,*B*). Following 10 DIV, we segmented probable varicosities, identified them based on morphology and dimensions, and evaluated the presence of Syt-1, a calcium sensor of exocytosis that is critical for neurotransmitter release in DA neurons (Mendez et al., 2011; Banerjee et al., 2020; Delignat-Lavaud et al., 2021), as an index of release-competent terminals. Strikingly, we find that vulnerable neurons have a significantly higher proportion of varicosities that are positive for Syt-1 (Fig. 8*A*,*B*; Extended Data Table 8-1), suggesting that these neurons have a higher proportion of active neurotransmitter release sites. Further examination of axonal varicosity density, calculated as intervaricosity distance and density per unit length of axon, revealed that there were no major differences between vulnerable and resilient neurons, with an intervaricose distance in the range ∼2–4 μm (Fig. 8*C*). Raphe serotonin and hypoglossal cholinergic neurons nonetheless had slightly higher intervaricose distances, while DMV and hypoglossal cholinergic neurons had a higher density of varicosities per unit length [Fig. 8*C*,*D*; unpaired mean difference of DMV (*n* = 12) minus SNc (*n* = 12) 45.6 [95CI 34.2; 72.2]] and XII neurons [unpaired mean difference of XII (*n* = 12) minus SNc (*n* = 12) 33.2 [95CI 24.8; 47.4]]. Together, these results provide support for the hypothesis that a distinguishing feature of vulnerable neurons is having an axonal arbor endowed with a high proportion of active neurotransmitter release sites, presumably linked with higher bioenergetic requirements, placing a larger load on the neuron’s mitochondrial network, that is equally dense across neuron types (Fig. 4*D*).

## Discussion

To our knowledge, this is the first study to compare the intrinsic vulnerability and morphologic characteristics of several neuronal populations suspected to be vulnerable in PD, to others considered as more resilient in this disorder. The results of this study show that neuronal populations considered vulnerable in PD, except for cholinergic DMV neurons, are more vulnerable to oxidative stress induced by hydrogen peroxide: a cellular stress model that is relevant in the context of the large body of work linking oxidative stress to cell loss in PD (Jenner, 2003; Monzani et al., 2019; Tokarew et al., 2021). We also find that vulnerable neurons are endowed with a broad axonal arbor that bears a higher proportion of Syt-1-positive axonal varicosities. Broadly, our work supports a model proposing that a large and highly arborized axonal domain, coupled with a dense network of active neurotransmitter release sites render projection neuromodulatory neurons vulnerable because of such characteristics being linked to a high mitochondrial-dependent bioenergetic burden and associated elevated sensitivity to extrahomeostatic conditions.

### Limitations

Some limitations of the present study need to be considered. We compared the growth of neurons obtained from different transgenic lines and expressing different fluorescent reporter proteins (GFP or TdTomato), which could possibly have influenced the vulnerability of the neurons examined. The growth of the different types of neurons examined under identical *in vitro* growth conditions and culture medium could have led to suboptimal growth for some of the neuronal populations, thus possibly influencing their intrinsic vulnerability and biasing the results. The dissection of some of the small nuclei examined could have also been imprecise and thus included small subsets of nonrelevant neurons of the same neurochemical identity. However, we have previously validated our dissections of small nuclei such as the VTA and SNc and our previously published confirmation of the differential size of their axonal arbor *in vitro* (Pacelli et al., 2015), like it is *in vivo* (Giguère et al., 2019), argues that our *in vitro* conditions were sufficient to allow at least some of the neurons’ intrinsic growth capacity to be maintained in culture.

### Neuronal populations considered as vulnerable in PD are more sensitive to cell stress induced by hydrogen peroxide

As hypothesized, neurons considered as more vulnerable in PD were as a class most sensitive to the hydrogen peroxide cell stress assay. This finding, and in particular the differential vulnerability of SNc and VTA DA neurons, is in line with previous work (Mosharov et al., 2009; Pacelli et al., 2015) demonstrating increased vulnerability of SNc DA neurons to both hydrogen peroxide and the mitochondrial toxin MPP+. The finding that cholinergic DMV neurons were relatively resilient to our neurotoxicity assay is somewhat surprising considering previous data suggesting that these neurons are affected in PD. However, it is also possible that DMV neurons are vulnerable in PD via mechanisms that are not directly related to oxidative stress, such as through ɑ-synuclein-dependent mechanisms (Chiu et al., 2021). However, given the very limited data derived from stereological counting methods validating whether DMV cholinergic neurons do, in fact, degenerate in PD (Giguère et al., 2018), it remains possible that our observation is explained by the fact that DMV cholinergic neurons are not particularly vulnerable in PD (Kalaitzakis et al., 2008). To strengthen these assertions, within the context of this experimental paradigm, it may be pertinent in future work to use other cellular stress assays, such as ɑ-synuclein overexpression and/or ɑ-synuclein fibrils.

### ROS production was not significantly different across neuron types

Using the mitochondrially-targeted ROS sensor mito-roGFP, we detected relatively similar levels of basal ROS production across all neuron types examined. We also found a very large spread of relative baseline oxidation status in the axonal and somatodendritic compartments of neurons (Fig. 4*C*). The lack of significant difference between neuron types may be a bit surprising considering that basal ROS production has been found to be significantly higher in SNc compared with VTA DA neurons both *in vitro* and *in vivo* (Guzman et al., 2010; Sanchez–Padilla et al., 2014; Pacelli et al., 2015), as well as in LC (Sanchez–Padilla et al., 2014) and DMV neurons (Goldberg et al., 2012). One possibility is that *in vitro*, the lack of a sufficient level of synaptic inputs severely limits the firing frequency of these neurons and thus the level of metabolic activity and energy needs. Additional experiments driving neuronal firing pharmacologically or optogenetically would be required to further test this hypothesis. The use of a cell-wide ROS sensor such as DHE, as previously used (Pacelli et al., 2015), would also help to assess ROS production deriving from other sources in addition to mitochondrial activity.

Strikingly, we also made the observation that, in our *in vitro* system, the distance between mitochondria along the axonal domain was consistent across neuron types (Fig. 4*D*). This observation suggests that vulnerable neurons with highly complex axonal arbors may have a density of mitochondria along their axons that is comparable to that of simpler, more resilient neurons. And therefore, their higher level of vulnerability may not simply derive from a higher density of mitochondria. It remains that given the differential vulnerability to hydrogen peroxide detected in the present study, it can be assumed that cell-autonomous developmental differences between these neurons are present in the *in vitro* postnatal culture model used.

### The complexity and activity of the axonal domain may be a key component of what renders a neuron vulnerable

A striking observation arising from the present study is the finding that globally, the neuronal populations that were most vulnerable to hydrogen peroxide neurotoxicity (SNc DA neurons, Raphe serotonin neurons and LC noradrenergic neurons) were also the ones with the largest axonal domain, and with the largest proportion of Syt-1-positive axonal varicosities. These findings are in line with the hypothesis that at least in part, the elevated vulnerability of long-range projection neuromodulatory neurons is linked to the very large energetic requirements of their large numbers of neurotransmitter release sites found along their vast axonal domain. This conclusion is compatible with the hypothesis that processes occurring in axon terminals, and in particular vesicular neurotransmitter packaging, is particularly costly for neurons in terms of energy requirements (Pulido and Ryan, 2021).

One relative “anomaly” in the present data set is the surprising resilience of cholinergic DMV neurons, especially considering their large axonal domain and large proportion of Syt-1-positive release sites. Perhaps, ChAT+ DMV neurons are significantly less vulnerable to hydrogen peroxide as they develop less complex, albeit very long, axonal arborizations. These arborization, in turn, contain far fewer varicosities per length of axon, despite having a similar proportion of varicosities that are active. Although we did not verify this in the present study, it can be reliably assumed that the presence of Syt-1 in varicosities is a reliable indicator of release site functionality (Brose et al., 1992; Ducrot et al., 2021). A lower proportion of Syt-1-positive axonal varicosities could result from several reasons, the simplest of which would be a lower relative level of expression of this gene. One possibility is that since DMV neurons project in large part to regions outside of the brain, the culture conditions used in the present study did not allow these neurons to get sufficient access to factors secreted by target cells in the peripheral nervous system and that are required to maintain these neurons’ excitability and normal baseline activity. Previous work has provided support for the role of target cells in regulating the proportion of axonal varicosities formed by SNc and VTA DA neurons (Ducrot et al., 2021). Furthermore, there is a vast literature on the role of extracellular signals regulating axonal development (Bilimoria and Bonni, 2013). Complementary electrophysiological recordings coupled to the use of genetically encoded sensors of activity-dependent vesicular cycling would help to examine this possibility further. It remains important to note that in the *in vitro* context used in the present study, all of the examined long-range projection neurons nonetheless developed a very large and branched axonal domain, orders of magnitude larger than most other neuron types under similar conditions, supporting the hypothesis that an intrinsic developmental program drives such exuberant axonal growth.

In conclusion, taken together, the results presented provide further support for the general relevance of the hypothesis that a key component of the selective vulnerability of neurons in PD (Wong et al., 2019) takes its origin in the large axonal domain of long-range projection neurons. We conclude that more efforts are now needed to better understand the bioenergetic challenges imposed by having a highly branched axonal domain endowed with a large number of active neurotransmitter release sites. In addition to the outright energetic demands imposed by such an axonal domain and by the firing of these neurons (Pacelli et al., 2015), it is intriguing to consider the possibility that such features may confer a massive demand on the endolysosomal system where, notably 12 out of 23 PARK genes (to denote their putative link to PD) are involved (Vidyadhara et al., 2019).

10.1523/ENEURO.0139-22.2022.tab6-1 Extended Data Table 6-1 Statistical reporting for Figure 6*B–D*. Download Table 6-1, DOCX file.

10.1523/ENEURO.0139-22.2022.tab8-1 Extended Data Table 8-1 Statistical reporting for Figure 8*B–D*. Download Table 8-1, DOCX file.

## Synthesis

Reviewing Editor: Mikko Airavaara, Helsingin yliopisto Decisions are customarily a result of the Reviewing Editor and the peer reviewers coming together and discussing their recommendations until a consensus is reached. When revisions are invited, a fact-based synthesis statement explaining their decision and outlining what is needed to prepare a revision will be listed below. The following reviewer(s) agreed to reveal their identity: NONE.

The paper eN-NWR-0139-22 explores the differential vulnerability of neurons to oxidative stress as it relates to Parkinson’s disease. To address this, the authors culture primary neurons from different regions of mouse brain and compare the response to oxidative stress caused by H2O2. Although the paper generated interesting data through the fragmentation analysis, the premise for paper and methods require additional information and revision before publication consideration by this reviewer.

1) The authors frame the paper in context of PD but this should be restricted to the Discussion. Although oxidative stress is clearly linked to PD, there are other mechanisms. The paper examines the vulnerability to oxidative stress for neurons from different mouse brain regions grown under identical conditions. The authors have some interesting observations and a revised manuscript that focuses on what was done and observed instead of trying to convince reader it was about PD may be more appropriate.

2) The differential vulnerability likely has little to do with “PD-vulnerable” but instead culture conditions in which each brain region is grown in. Given the relatively low density, their vulnerability under these conditions clearly won’t be same as in vivo where other support cells are present and in higher density. Also, different neurons respond more or less favorably depending on which culture media conditions, for example, neurotrophic factors such as GDNF for VM cultures has different outcome than cultures without GDNF. Were same reagents used for media as in the 2008 paper? A more detailed description of the plating and feeding of the cells is needed.

3) Why were different fluorescent proteins (GFP vs TdTomato) used? GFP has been associated with cellular toxicity. Also, does the presence of the fluorescent protein increase vulnerability to oxidative stress?

4) The authors are using post-natal tissue for practical reasons of culturing neurons but these are not the same as fully mature neurons in the brain regions of an adult organism where increased degeneration is observed. It is likely that some of the changes may be due to pre or post-natal exposure to oxidative stress that create long term consequences associated with variety of neurodegenerative diseases. Again, a revision in the claims of the paper more relevant to primary data are needed.

5) How are is the tissue dissected? They use a schematic that shows staining sections which of course aren’t stained. Do they use the fluorescent markers? Do they have images of before and after removal to show what was taken? The authors refer to 2008 paper that only focuses on the midbrain. Additional details and actual images of examples of separating the VTA vs SN should be shown (in mice this is often done with LCM). The schematic implies punches were taken but there is no mention of punches.

6) What were the TH-GFP mice used for? It seems they were only used in Figure 1? Why not use DAT-Cre x TdTomato (although this is a subset of TH).

7) No control virus was used for roGFP virus.

8) The presentation of statistics is confusing and disorganized in supplement. The authors should consider using tables rather than copy text from stat software. Also, please check the eNeuro guidelines for presenting statistics in separate table as well as implementing estimation statistics.

9) There is a reference to Figure 2C but there is no 2C. In general, the manuscript should be revised before resubmission making sure that Figures are complete and consistent. Units on axes should be unambiguous.

10) Genetic background can potentially affect neuronal properties (e.g. some Bl6 lines have αSyn deletion). Please, specify the exact genetic background of C57Bl6 mice in line 103 and in the description of all other mouse strains - have they all been maintained on the same genetic background?

11) Line 127: it is not clear, what kind of “slight modification” has been done in the current study in comparison to previously published protocol. Also, how many independent cultures have been prepared to obtain the data?

12) Line 298: In addition to LC neurons, TH promotor was probably also used to express mito-roGFP in SNc DA neurons. Please, clarify.

13) The authors use Syt1 immunostaining to analyze the number and density of neurotransmitter release sites. Could it be that the observed difference in Syt1-positive varicosities is due to different Syt1 levels in studied neuronal populations? In other words, can Syt1 be reliably used as a marker of neurotransmitter release sites across different neuronal populations? Please, comment.

14) In lines 370 and 385, the authors make rather strong statement that neuronal vulnerability can be explained by higher load on neuron’s mitochondrial network. However, their data demonstrate that, across studied neuronal types, mitochondrial density in similar (Fig. 4D) and so is the density of Syt1-positive varicosities (Fig. 8D). Moreover, there is no evidence of increased mitochondrial ROS production (Fig. 4C). Why do the authors conclude that there is larger load on the mitochondrial network? Rather, as also suggested by the authors in line 414, the differences in firing frequencies might be a crucial factor in neuronal vulnerability in PD. These points should be more clearly explained.

15) The authors state in lines 402-403 and 471 - studying αSyn overexpression and/or fibril-induced αSyn aggregation, as well as analyzing functions on endolysosomal system in the described experimental settings would be very interesting. It is encouraged that the authors to address these questions in follow-up studies.

16) Line 27: should read as “...PD-vulnerable neuronS are...”

17) Line 46: dopamine (DA) has already been abbreviated in line 43, no need to explain it again

18) Line 162: explain what “MTS” stands for - presumably, mitochondria localization signal?

19) Lines 163 and 165 - check superscript (-1) in titers, or simply state them as “vector genomes/ml"

20) Line 297: presumably, should read “... Cre recombinase-dependEnt...”

21) Line 505: “...redOx...”

22) Please, explain STD abbreviation in the Fig 4 legend.

23) Line 524: delete “... they different...”

24) Note the size of scale bars in figure legends.

25) Add the legend to graph on Fig 2B. What does grey group represent?

26) Fig. 3 - add Y-axis title to all graphs (presumably, the same as in Fig. 2B)

27) Fig. 5A - Why “SNc” is in red? In all other figures, the authors use blue color to denote “PD-vulnerable” neuronal types.

28) Fig. 8D - check Y-axis label; presumably, should read “Number of varicosities/axon length"

## Author Response

Response to reviewers

Reviewer #1:

1) The authors frame the paper in context of PD but this should be restricted to the Discussion.

Although oxidative stress is clearly linked to PD, there are other mechanisms. The paper examines the vulnerability to oxidative stress for neurons from different mouse brain regions grown under identical conditions. The authors have some interesting observations and a revised manuscript that focuses on what was done and observed instead of trying to convince reader it was about PD may be more appropriate.

Response: Our aims are not to over extrapolate the results we describe - rather describe some pertinent correlates of differential vulnerability of projecting neuromodulatory neuronal populations. Changes in the text have been made to address this point. The term “in Parkinson’s disease” has also been removed from the title. The new title of the manuscript would thus be:

"Axonal domain structure and function as a key cell-autonomous characteristic of selective vulnerability - a murine study of primary cultured neurons.” The terms PD-vulnerable, and PD-resilient have been changed to vulnerable and resilient, where appropriate.

2) The differential vulnerability likely has little to do with “PD-vulnerable” but instead culture conditions in which each brain region is grown in. Given the relatively low density, their vulnerability under these conditions clearly won’t be same as in vivo where other support cells are present and in higher density. Also, different neurons respond more or less favorably depending on which culture media conditions, for example, neurotrophic factors such as GDNF for VM cultures has different outcome than cultures without GDNF. Were same reagents used for media as in the 2008 paper? A more detailed description of the plating and feeding of the cells is needed.

Response: We agree that an in vitro cell culture system necessarily cannot claim to fully reproduce in vivo conditions and could certainly be missing a number of trophic and guidance signals that influence the growth and survival of the neurons that we examined. However, we have long-standing success in the growth of dopamine and other projection neurons. In the present project, we were also mainly interested in the intrinsic axonal growth characteristics of these neurons and their response to direct oxidative stress. Given that that we are comparing the survival of neurons that were cultured in identical conditions, we believe that we are able to make valuable comparisons. More information has been added to the methods to highlight that culture conditions and reagents were identical across target regions. We have also added a comment at the beginning of the discussion regarding the possible influence of missing growth factors.

3) Why were different fluorescent proteins (GFP vs TdTomato) used? GFP has been associated with cellular toxicity. Also, does the presence of the fluorescent protein increase vulnerability to oxidative stress?

Response: For most of the nuclei, we used a tdTomato reporter. However, to isolate the Locus Coeruleus, we were not able to locate a TH-Cre mouse. Instead, we used a TH-GFP mouse that we have used for more than 15 years and that has moderate levels of GFP expression and no issues of GFP-related toxicity. Otherwise, we only used GFP to detect oxidative stress using amodified redox-sensitive GFP (roGFP). This is by definition a construct that is sensitive to oxidative stress, but that by itself is not known to be toxic to neurons.

4) The authors are using post-natal tissue for practical reasons of culturing neurons but these are not the same as fully mature neurons in the brain regions of an adult organism where increased degeneration is observed. It is likely that some of the changes may be due to pre or post-natal exposure to oxidative stress that create long term consequences associated with variety of neurodegenerative diseases. Again, a revision in the claims of the paper more relevant to primary data are needed.

Response: We agree that cultured primary neurons cannot fully recapitulate the mature brain, just as iPSC-derived neurons increased used in PD research do not fully recapitulate the intact human brain. We agree with the reviewer that our in vitro primary culture system has many limitations. However, given that these neurons in our primary culture system have axonal arborizations totaling in the order of centimeters, we believe that comparing them to one another provides pertinent insights that contribute to our understanding of why they might, in the context of an aged human, have varying vulnerabilities. Passages in the text have been modified to reduce some of the claims made about the implications of these in vitro findings and some of the limitations of an in vitro system are discussed in the discussion section “The complexity and activity of the axonal domain may be a key component of what renders a neuron vulnerable”.

5) How are is the tissue dissected? They use a schematic that shows staining sections which of course aren’t stained. Do they use the fluorescent markers? Do they have images of before and after removal to show what was taken? The authors refer to 2008 paper that only focuses on the midbrain. Additional details and actual images of examples of separating the VTA vs SN should be shown (in mice this is often done with LCM). The schematic implies punches were taken but there is no mention of punches.

Response: We do not have images of the structures before and after dissection. This would be complicated because the dissections were not made from thin coronal sections for which a clean image before and after dissection could be obtained. We indeed used tdTomato fluorescence to guide the dissection of cholinergic neuron subgroups, 5-HT neurons and dopamine neurons. For locus ceoruleus neurons, we used GFP fluorescence as a guide. This is indicated in the transgenic animals section of the methods. Text has been added in the primary culture section of the methods to explain in more detail how this was done.

6) What were the TH-GFP mice used for? It seems they were only used in Figure 1? Why not use DAT-Cre x TdTomato (although this is a subset of TH).

Response: TH-GFP mice were used to accurately target TH+ (noradrenergic) neurons of the LC.

These neurons do not express the DAT and therefore are not labelled in DAT-Cre/Ai9 mice.

7) No control virus was used for roGFP virus.

Response: Correct. The roGFP construct was used specifically to estimate oxidative stress levels.

It was not used as a treatment that would have required a control. Given that we are comparing all neurons that are expressing roGFP, any confound of the expression is assumed to be present in all neurons.

8) The presentation of statistics is confusing and disorganized in supplement. The authors should consider using tables rather than copy text from stat software. Also, please check the eNeuro guidelines for presenting statistics in separate table as well as implementing estimation statistics.

Response: We did use estimation statistic. The initial supplement was an R markdown document, enabling reproduction of the analysis from derived raw data. But we nonetheless agree that a table format might be easier for reader to understand. We have now done this in the revised manuscript.

9) There is a reference to Figure 2C but there is no 2C. In general, the manuscript should be revised before resubmission making sure that Figures are complete and consistent. Units on axes should be unambiguous.

Response: This has been addressed in the revised manuscript.

10) Genetic background can potentially affect neuronal properties (e.g. some Bl6 lines have αSyn deletion). Please, specify the exact genetic background of C57Bl6 mice in line 103 and in the description of all other mouse strains - have they all been maintained on the same genetic background?

Response: We have added the requested details on the mouse genetic background in the methods section.

11) Line 127: it is not clear, what kind of “slight modification” has been done in the current study in comparison to previously published protocol. Also, how many independent cultures have been prepared to obtain the data?

Response: The slight modifications imply the difference in target structures, and changes in sourcing of exact media (supplier). All experiments were performed on at least 3 (3-6) independent cultures (in text - Primary Cell Culture methods).

12) Line 298: In addition to LC neurons, TH promotor was probably also used to express mito-roGFP in SNc DA neurons. Please, clarify.

Response: To express the MTS-roGRP construct in dopamine neurons, we used the AAV9-CMV-DIO-MTS-roGFP-WPRE-bGHpA virus, which is a cre-dependent construct. These experiments were performed in dopamine neurons from DAT-IRES-cre animals. This is specified in the methods sections “Mitochondrial targeting of the ROS sensor, roGFP"

13) The authors use Syt1 immunostaining to analyze the number and density of neurotransmitter release sites. Could it be that the observed difference in Syt1-positive varicosities is due to different Syt1 levels in studied neuronal populations? In other words, can Syt1 be reliably used as a marker of neurotransmitter release sites across different neuronal populations? Please, comment.

Response: This is a fair comment. We agree that syt1 immunostaining as a proxy for active neurotransmitter release site is not without shortcomings. However, if a neuronal population has a lower proportion of Syt1-positive terminals because its level of Syt1 expression is lower, this leads to the same conclusion, i.e., less active terminals. We have now added a sentence in the revised discussion (page 23) to discuss this point.

14) In lines 370 and 385, the authors make rather strong statement that neuronal vulnerability can be explained by higher load on neuron’s mitochondrial network. However, their data demonstrate that, across studied neuronal types, mitochondrial density in similar (Fig. 4D) and so is the density of Syt1-positive varicosities (Fig. 8D). Moreover, there is no evidence of increased mitochondrial ROS production (Fig. 4C). Why do the authors conclude that there is larger load on the mitochondrial network? Rather, as also suggested by the authors in line 414, the differences in firing frequencies might be a crucial factor in neuronal vulnerability in PD. These points should be more clearly explained.

Response: The main reason to think that there is a larger load on mitochondria in vulnerable neurons is that these neurons are likely to be endowed with a higher number of active neurotransmitter release sites. So, even if along the axon, the density of mitochondria is similar in the different types of neurons, these mitochondria would end up being more solicited by neurons with a higher proportion of active terminals. But we certainly also agree that if this energetic load is related to the activity of the terminals and to vesicular packaging and cycling, the neurons’ firing rates would also play a key role. Compatible with this model, we previously showed that blocking firing decreased oxygen consumption by dopamine neurons (Pacelli et al., 2015). We have now added a reference to the role of firing in the concluding statements (page 24).

15) The authors state in lines 402-403 and 471 - studying αSyn overexpression and/or fibril-induced αSyn aggregation, as well as analyzing functions on endolysosomal system in the described experimental settings would be very interesting. It is encouraged that the authors to address these questions in follow-up studies.

Response: We will. Thanks.

16) Line 27: should read as “...PD-vulnerable neuronS are...”

Response: Done

17) Line 46: dopamine (DA) has already been abbreviated in line 43, no need to explain it again

Response: Done

18) Line 162: explain what “MTS” stands for - presumably, mitochondria localization signal?

Response: This has been added to methods section (page 9).

19) Lines 163 and 165 - check superscript (-1) in titers, or simply state them as “vector genomes/ml"

Response: This has been modified.

20) Line 297: presumably, should read “... Cre recombinase-dependEnt...”

Response: This has been modified.

21) Line 505: “...redOx...”

Response: This has been corrected.

22) Please, explain STD abbreviation in the Fig 4 legend.

Response: This means “somatodendritic compartment (STD)"

23) Line 524: delete “... they different...”

Response: This has been modified.

24) Note the size of scale bars in figure legends.

Response: All scale bar sizes are described in figures.

25) Add the legend to graph on Fig 2B. What does grey group represent?

Response: These boxes were added to aid in visualizing the different groups - 0, 100, 150, 200. We have now removed them.

26) Fig. 3 - add Y-axis title to all graphs (presumably, the same as in Fig. 2B)

Response: The Y-axis label is on the left-hand side of the figure -> “Normalized number of positive neurons"

27) Fig. 5A - Why “SNc” is in red? In all other figures, the authors use blue color to denote “PD-vulnerable” neuronal types.

Response: Thanks for pointing this out. This was an error - it has been corrected.

28) Fig. 8D - check Y-axis label; presumably, should read “Number of varicosities/axon length"

Response: Correct, the modification has been made.

## References

[B1] Alberico SL, Cassell MD, Narayanan NS (2015) The vulnerable ventral tegmental area in Parkinson’s disease. Basal Ganglia 5:51–55. 10.1016/j.baga.2015.06.001 26251824 PMC4523275

[B2] Allen Brain Atlas (2008) ISH data :: Allen Brain Atlas: developing mouse brain [WWW document]. Available at https://developingmouse.brain-map.org/. Accessed June 4, 2021.

[B100] Bäckman CM, Malik N, Zhang Y, Shan L, Grinberg A, Hoffer BJ, Westphal H, Tomac AC (2006) Characterization of a mouse strain expressing Cre recombinase from the 3’ untranslated region of the dopamine transporter locus. Genesis 44:383–390. 16865686 10.1002/dvg.20228

[B3] Banerjee A, Lee J, Nemcova P, Liu C, Kaeser PS (2020) Synaptotagmin-1 is the Ca2+ sensor for fast striatal dopamine release. Elife 9:e58359. 10.7554/eLife.58359 32490813 PMC7319770

[B4] Bilimoria PM, Bonni A (2013) Molecular control of axon branching. Neuroscientist 19:16–24. 10.1177/1073858411426201 22179123 PMC3490022

[B5] Bolam JP, Pissadaki EK (2012) Living on the edge with too many mouths to feed: why dopamine neurons die. Mov Disord 27:1478–1483. 10.1002/mds.25135 23008164 PMC3504389

[B6] Brose N, Petrenko AG, Südhof TC, Jahn R (1992) Synaptotagmin: a calcium sensor on the synaptic vesicle surface. Science 256:1021–1025. 10.1126/science.1589771 1589771

[B7] Canadian Council on Animal Care (1993) Guide to the care and use of experimental animals. Vol. 1. Ottawa: Canadian Council on Animal Care.

[B8] Chiu WH, Kovacheva L, Musgrove RE, Arien-Zakay H, Koprich JB, Brotchie JM, Yaka R, Ben-Zvi D, Hanani M, Roeper J, Goldberg JA (2021) α-Synuclein–induced Kv4 channelopathy in mouse vagal motoneurons drives nonmotor parkinsonian symptoms. Sci Adv 7:eabd3994. 33692101 10.1126/sciadv.abd3994 PMC7946367

[B9] Delignat-Lavaud B, Kano J, Ducrot C, Massé I, Mukherjee S, Giguère N, Moquin L, Lévesque C, Nanni SB, Bourque M-J, Rosa-Neto P, Lévesque D, Beaumont LD, Trudeau L-É (2021) The calcium sensor synaptotagmin-1 is critical for phasic axonal dopamine release in the striatum and mesencephalon, but is dispensable for basic motor behaviors in mice. bioRxiv 460511. doi:10.1101/2021.09.15.460511. 10.1038/s41467-023-39805-7 PMC10336101 37433762

[B10] Ducrot C, Bourque MJ, Delmas CVL, Racine AS, Guadarrama Bello D, Delignat-Lavaud B, Domenic Lycas M, Fallon A, Michaud-Tardif C, Burke Nanni S, Herborg F, Gether U, Nanci A, Takahashi H, Parent M, Trudeau LE (2021) Dopaminergic neurons establish a distinctive axonal arbor with a majority of non-synaptic terminals. FASEB J 35:e21791. 34320240 10.1096/fj.202100201RR

[B12] Espay AJ, Lang AE (2018) Parkinson diseases in the 2020s and beyond: replacing clinico-pathologic convergence with systems biology divergence. J Park Dis 8:S59–S64. 10.3233/JPD-181465 PMC6311362 30584155

[B13] Fasano C, Thibault D, Trudeau LÉ (2008) Culture of postnatal mesencephalic dopamine neurons on an astrocyte monolayer. Curr Protoc Neurosci 44:3.21.1–3.21.19. 10.1002/0471142301.ns0321s44 18633997

[B14] Giguère N, Burke Nanni S, Trudeau LE (2018) On cell loss and selective vulnerability of neuronal populations in Parkinson’s disease. Front Neurol 9:455. 10.3389/fneur.2018.00455 29971039 PMC6018545

[B15] Giguère N, Delignat-Lavaud B, Herborg F, Voisin A, Li Y, Jacquemet V, Anand-Srivastava M, Gether U, Giros B, Trudeau LÉ (2019) Increased vulnerability of nigral dopamine neurons after expansion of their axonal arborization size through D2 dopamine receptor conditional knockout. PLoS Genet 15:e1008352. 10.1371/journal.pgen.1008352 31449520 PMC6730950

[B16] Goedert M, Spillantini MG, Del Tredici K, Braak H (2013) 100 years of Lewy pathology. Nat Rev Neurol 9:13–24. 10.1038/nrneurol.2012.242 23183883

[B17] Goldberg JA, Guzman JN, Estep CM, Ilijic E, Kondapalli J, Sanchez-Padilla J, Surmeier DJ (2012) Calcium entry induces mitochondrial oxidant stress in vagal neurons at risk in Parkinson’s disease. Nat Neurosci 15:1414–1421. 10.1038/nn.3209 22941107 PMC3461271

[B18] Gong S, Doughty M, Harbaugh CR, Cummins A, Hatten ME, Heintz N, Gerfen CR (2007) Targeting Cre recombinase to specific neuron populations with bacterial artificial chromosome constructs. J Neurosci 27:9817–9823. 10.1523/JNEUROSCI.2707-07.2007 17855595 PMC6672645

[B19] Guzman JN, Sanchez-Padilla J, Wokosin D, Kondapalli J, Ilijic E, Schumacker PT, Surmeier DJ (2010) Oxidant stress evoked by pacemaking in dopaminergic neurons is attenuated by DJ-1. Nature 468:696–700. 10.1038/nature09536 21068725 PMC4465557

[B20] Hirsch EC, Graybiel AM, Duyckaerts C, Javoy-Agid F (1987) Neuronal loss in the pedunculopontine tegmental nucleus in Parkinson disease and in progressive supranuclear palsy. Proc Natl Acad Sci U S A 84:5976–5980. 10.1073/pnas.84.16.5976 3475716 PMC298986

[B21] Ho JW, Tumkaya T (2020) Data analysis using bootstrap-coupled estimation [R package dabestr version 0.3.0] [WWW document]. Available at https://CRAN.R-project.org/package=dabestr. Accessed June 3, 2021.

[B22] Ho J, Tumkaya T, Aryal S, Choi H, Claridge-Chang A (2019) Moving beyond p values: data analysis with estimation graphics. Nat Methods 16:565–566. 10.1038/s41592-019-0470-3 31217592

[B23] Huynh B, Fu Y, Kirik D, Shine JM, Halliday GM (2021) Comparison of locus coeruleus pathology with nigral and forebrain pathology in Parkinson’s disease. Mov Disord 36:2085–2093. 33899954 10.1002/mds.28615

[B24] Jenner P (2003) Oxidative stress in Parkinson’s disease. Ann Neurol 53:S26–S38. 10.1002/ana.10483 12666096

[B25] Kalaitzakis ME, Graeber MB, Gentleman SM, Pearce RKB (2008) The dorsal motor nucleus of the vagus is not an obligatory trigger site of Parkinson’s disease: a critical analysis of alpha-synuclein staging. Neuropathol Appl Neurobiol 34:284–295. 10.1111/j.1365-2990.2007.00923.x 18053026

[B26] Lehtonen Š, Sonninen T-M, Wojciechowski S, Goldsteins G, Koistinaho J (2019) Dysfunction of cellular proteostasis in Parkinson’s disease. Front Neurosci 13:457. 31133790 10.3389/fnins.2019.00457 PMC6524622

[B27] Lein ES, Hawrylycz MJ, Ao N, Ayres M, Bensinger A, Bernard A, Boe AF, Boguski MS, Brockway KS, Byrnes EJ, Chen L, Chen L, Chen TM, Chin MC, Chong J, Crook BE, Czaplinska A, Dang CN, Datta S, Dee NR, et al. (2007) Genome-wide atlas of gene expression in the adult mouse brain. Nature 445:168–176. 10.1038/nature05453 17151600

[B28] Lieberman OJ, Choi SJ, Kanter E, Saverchenko A, Frier MD, Fiore GM, Wu M, Kondapalli J, Zampese E, Surmeier DJ, Sulzer D, Mosharov EV (2017) α-Synuclein-dependent calcium entry underlies differential sensitivity of cultured SN and VTA dopaminergic neurons to a Parkinsonian neurotoxin. eNeuro 4:ENEURO.0167-17.2017. 10.1523/ENEURO.0167-17.2017 PMC5701296 29177188

[B29] Lunt W, Shah M, Sang J, Choi J, Burke S, Higgins J, Skene N (2021) Neurochemical identity and anatomy of cell loss in Parkinson’s disease, and the clinical data available for correlation of progression: a systematic review and meta-analysis. PROSPERO 2021.

[B101] Madisen L, Zwingman TA, Sunkin SM, Oh SW, Zariwala HA, Gu H, Ng LL, Palmiter RD, Hawrylycz MJ, Jones AR, Lein ES, Zeng H (2010) A robust and high-throughput Cre reporting and characterization system for the whole mouse brain. Nat Neurosci 13:133–140. 20023653 10.1038/nn.2467 PMC2840225

[B30] Matsushita N, Okada H, Yasoshima Y, Takahashi K, Kiuchi K, Kobayashi K (2002) Dynamics of tyrosine hydroxylase promoter activity during midbrain dopaminergic neuron development. J Neurochem 82:295–304. 10.1046/j.1471-4159.2002.00972.x 12124430

[B31] Mendez JA, Bourque M-J, Fasano C, Kortleven C, Trudeau L-E (2011) Somatodendritic dopamine release requires synaptotagmin 4 and 7 and the participation of voltage-gated calcium channels. J Biol Chem 286:23928–23937. 10.1074/jbc.M111.218032 21576241 PMC3129174

[B32] Monzani E, Nicolis S, Dell’Acqua S, Capucciati A, Bacchella C, Zucca FA, Mosharov EV, Sulzer D, Zecca L, Casella L (2019) Dopamine, oxidative stress and protein–quinone modifications in Parkinson’s and other neurodegenerative diseases. Angew Chem Int Ed Engl 58:6512–6527. 10.1002/anie.201811122 30536578

[B33] Mosharov EV, Larsen KE, Kanter E, Phillips KA, Wilson K, Schmitz Y, Krantz DE, Kobayashi K, Edwards RH, Sulzer D (2009) Interplay between cytosolic dopamine, calcium, and alpha-synuclein causes selective death of substantia nigra neurons. Neuron 62:218–229. 10.1016/j.neuron.2009.01.033 19409267 PMC2677560

[B34] Müller K (2020) A simpler way to find your files [R package here version 1.0.1] [WWW document]. Available at https://CRAN.R-project.org/package=here. Accessed June 3, 2021.

[B35] Ogle D, Doll J, Wheeler P, Dinno A (2021) FSA: simple fisheries stock assessment methods [r package fsa version 0. 8. 32] [WWW document]. Available at https://cran.r-project.org/web/packages/FSA/citation.html. Accessed June 3, 2021.

[B36] Pacelli C, Giguère N, Bourque M-J, Lévesque M, Slack RS, Trudeau L-É (2015) Elevated mitochondrial bioenergetics and axonal arborization size are key contributors to the vulnerability of dopamine neurons. Curr Biol 25:2349–2360. 10.1016/j.cub.2015.07.050 26320949

[B37] Parent M, Parent A (2006) Single-axon tracing study of corticostriatal projections arising from primary motor cortex in primates. J Comp Neurol 496:202–213. 10.1002/cne.20925 16538675

[B38] Pedersen TL (2021) Accelerating “ggplot2” [R package ggforce version 0.3.3] [WWW document]. Available at https://CRAN.R-project.org/package=ggforce. Accessed June 3, 2021.

[B39] Pissadaki EK, Bolam JP (2013) The energy cost of action potential propagation in dopamine neurons: clues to susceptibility in Parkinson’s disease. Front Comput Neurosci 7:13. 10.3389/fncom.2013.00013 23515615 PMC3600574

[B40] Pulido C, Ryan TA (2021) Synaptic vesicle pools are a major hidden resting metabolic burden of nerve terminals. Sci Adv 7:eabi9027. 10.1126/sciadv.abi9027 34860552 PMC8641928

[B41] R Core Team (2017) R: a language and environment for statistical computing. Vienna: R Foundation for Statistical Computing.

[B42] Rossi J, Balthasar N, Olson D, Scott M, Berglund E, Lee CE, Choi MJ, Lauzon D, Lowell BB, Elmquist JK (2011) Melanocortin-4 receptors expressed by cholinergic neurons regulate energy balance and glucose homeostasis. Cell Metab 13:195–204. 10.1016/j.cmet.2011.01.010 21284986 PMC3033043

[B43] RStudio Team (2020) RStudio: Integrated Development for R. RStudio, PBC, Boston, MA. Available at http://www.rstudio.com/.

[B44] Sanchez–Padilla J, Guzman JN, Ilijic E, Kondapalli J, Galtieri DJ, Yang B, Schieber S, Oertel W, Wokosin D, Schumacker PT, Surmeier DJ (2014) Mitochondrial oxidant stress in locus coeruleus is regulated by activity and nitric oxide synthase. Nat Neurosci 17:832–840. 10.1038/nn.3717 24816140 PMC4131291

[B45] Schneider CA, Rasband WS, Eliceiri KW (2012) NIH Image to ImageJ: 25 years of image analysis. Nat Methods 9:671–675. 10.1038/nmeth.2089 22930834 PMC5554542

[B46] Slowikowski K (2021) Automatically position non-overlapping text labels with “ggplot2” [R package ggrepel version 0.9.1] [WWW document]. Available at https://CRAN.R-project.org/package=ggrepel. Accessed June 3, 2021.

[B47] Sofroniew N, et al. (2021) napari/napari: 0.4.8rc2. Zenodo. Available at https://zenodo.org/record/4747712#.Yzq_fHZByUk0.

[B48] Surmeier DJ, Obeso JA, Halliday GM (2017a) Parkinson’s disease is not simply a prion disorder. J Neurosci 37:9799–9807. 10.1523/JNEUROSCI.1787-16.2017 29021297 PMC5637112

[B49] Surmeier DJ, Obeso JA, Halliday GM (2017b) Selective neuronal vulnerability in Parkinson disease. Nat Rev Neurosci 18:101–113. 10.1038/nrn.2016.178 28104909 PMC5564322

[B51] Thévenaz P, Ruttimann UE, Unser M (1998) A pyramid approach to subpixel registration based on intensity. IEEE Trans Image Process 7:27–41. 10.1109/83.650848 18267377

[B11] Tokarew JM, et al. (2021) Age-associated insolubility of parkin in human midbrain is linked to redox balance and sequestration of reactive dopamine metabolites. Acta Neuropathol 141:725–754. 33694021 10.1007/s00401-021-02285-4 PMC8043881

[B52] Tubert C, Galtieri D, Surmeier DJ (2019) Ten years of experience of pedunculopontine nucleus deep brain stimulation: a reappraisal from the basic to the clinic. Neurobiol Dis 128:3–8. 30171892 10.1016/j.nbd.2018.08.017 PMC6546542

[B53] Vidyadhara DJ, Lee JE, Chandra SS (2019) Role of the endolysosomal system in Parkinson’s disease. J Neurochem 150:487–506. 10.1111/jnc.14820 31287913 PMC6707858

[B54] Wickham H (2021) Tidy messy data [R package tidyr version 1.1.3] [WWW document]. Available at https://CRAN.R-project.org/package=tidyr. Accessed June 3, 2021.

[B55] Wilke CO (2021) Ridgeline plots in “ggplot2” [R package ggridges version 0.5.3] [WWW document]. Available at https://CRAN.R-project.org/package=ggridges. Accessed June 3, 2021.

[B56] Wong YC, Luk K, Purtell K, Burke Nanni S, Stoessl AJ, Trudeau L, Yue Z, Krainc D, Oertel W, Obeso JA, Volpicelli‐Daley LA (2019) Neuronal vulnerability in Parkinson disease: should the focus be on axons and synaptic terminals? Mov Disord 34:1406–1422. 10.1002/mds.27823 31483900 PMC6879792

