# Supplementary material for: Axonal Domain Structure as a Putative Identifier of Neuron-Specific Vulnerability to Oxidative Stress in Cultured Neurons
Source: eNeuro. 2022 Oct 24;9(5):ENEURO.0139-22.2022. doi: 10.1523/ENEURO.0139-22.2022 (PMC9595591; doi:10.1523/ENEURO.0139-22.2022)
Supplement: Extended Data Table 2-1 — Statistical reporting for Figure 2B. Download Table 2-1, DOCX file. [file enu-eN-NWR-0139-22-s03.docx]

**EXTENDED TABLE FOR FIGURE 2B**

Kurskal-Wallis

Kruskal-Wallis rank sum test

data: neuron_normalized by hydrogen_peroxide
Kruskal-Wallis chi-squared = 164.32, df = 3, p-value < 2.2e-16

Dunn test

| Comparison | Z | P.unadj | P.adj |
| --- | --- | --- | --- |
| 0 - 100 | 5.867938 | 0.0000000 | 0.0000000 |
| 0 - 150 | 9.779527 | 0.0000000 | 0.0000000 |
| 100 - 150 | 3.408957 | 0.0006521 | 0.0039127 |
| 0 - 200 | 11.754230 | 0.0000000 | 0.0000000 |
| 100 - 200 | 5.563542 | 0.0000000 | 0.0000002 |
| 150 - 200 | 2.386850 | 0.0169934 | 0.1019605 |
