# Supplementary material for: Axonal Domain Structure as a Putative Identifier of Neuron-Specific Vulnerability to Oxidative Stress in Cultured Neurons
Source: eNeuro. 2022 Oct 24;9(5):ENEURO.0139-22.2022. doi: 10.1523/ENEURO.0139-22.2022 (PMC9595591; doi:10.1523/ENEURO.0139-22.2022)
Supplement: Extended Data Table 3-1 — Statistical reporting for Figure 3A–D. Download Table 3-1, DOCX file. [file enu-eN-NWR-0139-22-s04.docx]

**EXTENDED TABLE FOR FIGURE 3A**

Kruskal-Wallis and Dunn test for each concertation

**0 micromollar**

Kruskal-Wallis rank sum test

data: neuron_normalized by neuron
Kruskal-Wallis chi-squared = 0.43515, df = 6, p-value = 0.9985

| Comparison | Z | P.unadj | P.adj |
| --- | --- | --- | --- |
| DMV - LC | 0.0460099 | 0.9633024 | 1 |
| DMV - R | -0.0170668 | 0.9863833 | 1 |
| LC - R | -0.0570271 | 0.9545236 | 1 |
| DMV - SN | 0.0596846 | 0.9524069 | 1 |
| LC - SN | 0.0120472 | 0.9903880 | 1 |
| R - SN | 0.0686879 | 0.9452381 | 1 |
| DMV - STR | 0.3880572 | 0.6979737 | 1 |
| LC - STR | 0.3587423 | 0.7197879 | 1 |
| R - STR | 0.3499389 | 0.7263845 | 1 |
| SN - STR | 0.3661004 | 0.7142902 | 1 |
| DMV - VTA | 0.3929825 | 0.6943324 | 1 |
| LC - VTA | 0.3644469 | 0.7155243 | 1 |
| R - VTA | 0.3573996 | 0.7207927 | 1 |
| SN - VTA | 0.3708062 | 0.7107819 | 1 |
| STR - VTA | 0.0223996 | 0.9821292 | 1 |
| DMV - XII | 0.3419833 | 0.7323635 | 1 |
| LC - XII | 0.3099783 | 0.7565775 | 1 |
| R - XII | 0.3087642 | 0.7575009 | 1 |
| SN - XII | 0.3153817 | 0.7524719 | 1 |
| STR - XII | -0.0591645 | 0.9528211 | 1 |
| VTA - XII | -0.0792485 | 0.9368350 | 1 |

**EXTENDED TABLE FOR FIGURE 3B**

**100 micromollar**

Kruskal-Wallis rank sum test

data: neuron_normalized by neuron
Kruskal-Wallis chi-squared = 27.496, df = 6, p-value = 0.0001169

| Comparison | Z | P.unadj | P.adj |
| --- | --- | --- | --- |
| DMV - LC | 3.0576763 | 0.0022306 | 0.0468427 |
| DMV - R | 1.3473497 | 0.1778676 | 1.0000000 |
| LC - R | -0.9882252 | 0.3230424 | 1.0000000 |
| DMV - SN | 2.1096460 | 0.0348889 | 0.7326661 |
| LC - SN | -0.9597891 | 0.3371614 | 1.0000000 |
| R - SN | 0.2807619 | 0.7788930 | 1.0000000 |
| DMV - STR | -1.8377294 | 0.0661023 | 1.0000000 |
| LC - STR | -4.7762384 | 0.0000018 | 0.0000375 |
| R - STR | -2.8157629 | 0.0048662 | 0.1021893 |
| SN - STR | -3.8739054 | 0.0001071 | 0.0022492 |
| DMV - VTA | 1.1366643 | 0.2556787 | 1.0000000 |
| LC - VTA | -1.7571423 | 0.0788936 | 1.0000000 |
| R - VTA | -0.3995113 | 0.6895165 | 1.0000000 |
| SN - VTA | -0.8624297 | 0.3884511 | 1.0000000 |
| STR - VTA | 2.8668055 | 0.0041464 | 0.0870739 |
| DMV - XII | 1.2946210 | 0.1954510 | 1.0000000 |
| LC - XII | -1.9376752 | 0.0526629 | 1.0000000 |
| R - XII | -0.3945921 | 0.6931439 | 1.0000000 |
| SN - XII | -0.9231033 | 0.3559534 | 1.0000000 |
| STR - XII | 3.1542542 | 0.0016091 | 0.0337909 |
| VTA - XII | 0.0389910 | 0.9688976 | 1.0000000 |

**EXTENDED TABLE FOR FIGURE 3C**

**150 micromollar**

Kruskal-Wallis rank sum test

data: neuron_normalized by neuron
Kruskal-Wallis chi-squared = 43.006, df = 6, p-value = 1.163e-07

| Comparison | Z | P.unadj | P.adj |
| --- | --- | --- | --- |
| DMV - LC | 3.4092318 | 0.0006515 | 0.0136807 |
| DMV - R | 1.8603390 | 0.0628376 | 1.0000000 |
| LC - R | -0.9981418 | 0.3182106 | 1.0000000 |
| DMV - SN | 4.5926293 | 0.0000044 | 0.0000919 |
| LC - SN | 1.1229937 | 0.2614402 | 1.0000000 |
| R - SN | 1.9281774 | 0.0538331 | 1.0000000 |
| DMV - STR | -0.9114641 | 0.3620509 | 1.0000000 |
| LC - STR | -3.8608477 | 0.0001130 | 0.0023729 |
| R - STR | -2.4791760 | 0.0131686 | 0.2765412 |
| SN - STR | -4.8615781 | 0.0000012 | 0.0000245 |
| DMV - VTA | 2.0924238 | 0.0364006 | 0.7644130 |
| LC - VTA | -1.0786649 | 0.2807371 | 1.0000000 |
| R - VTA | 0.0290625 | 0.9768148 | 1.0000000 |
| SN - VTA | -2.1280995 | 0.0333288 | 0.6999055 |
| STR - VTA | 2.7134661 | 0.0066583 | 0.1398251 |
| DMV - XII | 0.5206388 | 0.6026184 | 1.0000000 |
| LC - XII | -3.0994014 | 0.0019391 | 0.0407215 |
| R - XII | -1.5027565 | 0.1329018 | 1.0000000 |
| SN - XII | -4.3683599 | 0.0000125 | 0.0002629 |
| STR - XII | 1.3973720 | 0.1623017 | 1.0000000 |
| VTA - XII | -1.7165660 | 0.0860585 | 1.0000000 |

**EXTENDED TABLE FOR FIGURE 3D**

**200 micromollar**

Kruskal-Wallis rank sum test

data: neuron_normalized by neuron
Kruskal-Wallis chi-squared = 55.538, df = 6, p-value = 3.608e-10

| Comparison | Z | P.unadj | P.adj |
| --- | --- | --- | --- |
| DMV - LC | 4.6306976 | 0.0000036 | 0.0000765 |
| DMV - R | 1.7772743 | 0.0755231 | 1.0000000 |
| LC - R | -1.7758038 | 0.0757653 | 1.0000000 |
| DMV - SN | 3.6600377 | 0.0002522 | 0.0052957 |
| LC - SN | -0.8816647 | 0.3779581 | 1.0000000 |
| R - SN | 1.0949050 | 0.2735583 | 1.0000000 |
| DMV - STR | -0.9378490 | 0.3483220 | 1.0000000 |
| LC - STR | -5.2402220 | 0.0000002 | 0.0000034 |
| R - STR | -2.4707312 | 0.0134837 | 0.2831579 |
| SN - STR | -4.3427853 | 0.0000141 | 0.0002954 |
| DMV - VTA | 0.2734029 | 0.7845435 | 1.0000000 |
| LC - VTA | -4.3311997 | 0.0000148 | 0.0003114 |
| R - VTA | -1.5540418 | 0.1201745 | 1.0000000 |
| SN - VTA | -3.3718458 | 0.0007467 | 0.0156799 |
| STR - VTA | 1.1909709 | 0.2336650 | 1.0000000 |
| DMV - XII | -0.0642890 | 0.9487401 | 1.0000000 |
| LC - XII | -5.0075611 | 0.0000006 | 0.0000116 |
| R - XII | -1.8911995 | 0.0585977 | 1.0000000 |
| SN - XII | -3.9502450 | 0.0000781 | 0.0016395 |
| STR - XII | 0.9217728 | 0.3566471 | 1.0000000 |
| VTA - XII | -0.3524809 | 0.7244776 | 1.0000000 |

**Estimation statistics**

**EXTENDED TABLE FOR FIGURE 3A**

**0 micromollar**

| control_group | test_group | difference | bca_ci_low | bca_ci_high |
| --- | --- | --- | --- | --- |
| SN | VTA | 0.000 | -0.179 | 0.212 |
| SN | LC | 0.000 | -0.168 | 0.160 |
| SN | R | 0.000 | -0.175 | 0.160 |
| SN | DMV | 0.007 | -0.155 | 0.160 |
| SN | XII | 0.000 | -0.237 | 0.238 |
| SN | STR | 0.030 | -0.174 | 0.259 |

**EXTENDED TABLE FOR FIGURE 3B**

**100 micromollar**

| control_group | test_group | difference | bca_ci_low | bca_ci_high |
| --- | --- | --- | --- | --- |
| SN | VTA | 0.061 | -0.142 | 0.260 |
| SN | LC | -0.084 | -0.213 | 0.052 |
| SN | R | 0.031 | -0.119 | 0.187 |
| SN | DMV | 0.197 | 0.066 | 0.340 |
| SN | XII | 0.152 | -0.044 | 0.388 |
| SN | STR | 0.581 | 0.350 | 0.831 |

**EXTENDED TABLE FOR FIGURE 3C**

**150 micromollar**

| control_group | test_group | difference | bca_ci_low | bca_ci_high |
| --- | --- | --- | --- | --- |
| SN | VTA | 0.182 | -0.031 | 0.390 |
| SN | LC | 0.068 | -0.092 | 0.188 |
| SN | R | 0.159 | -0.032 | 0.311 |
| SN | DMV | 0.353 | 0.192 | 0.478 |
| SN | XII | 0.388 | 0.200 | 0.595 |
| SN | STR | 0.523 | 0.308 | 0.734 |

**EXTENDED TABLE FOR FIGURE 3D**

**200 micromollar**

| control_group | test_group | difference | bca_ci_low | bca_ci_high |
| --- | --- | --- | --- | --- |
| SN | VTA | 0.387 | 0.139 | 0.611 |
| SN | LC | -0.105 | -0.268 | -0.011 |
| SN | R | 0.024 | -0.151 | 0.163 |
| SN | DMV | 0.342 | 0.146 | 0.507 |
| SN | XII | 0.365 | 0.170 | 0.534 |
| SN | STR | 0.585 | 0.322 | 0.827 |
