# Supplementary figures and images for: Axonal Domain Structure as a Putative Identifier of Neuron-Specific Vulnerability to Oxidative Stress in Cultured Neurons
Source: eNeuro. 2022 Oct 24;9(5):ENEURO.0139-22.2022. doi: 10.1523/ENEURO.0139-22.2022 (PMC9595591; doi:10.1523/ENEURO.0139-22.2022)

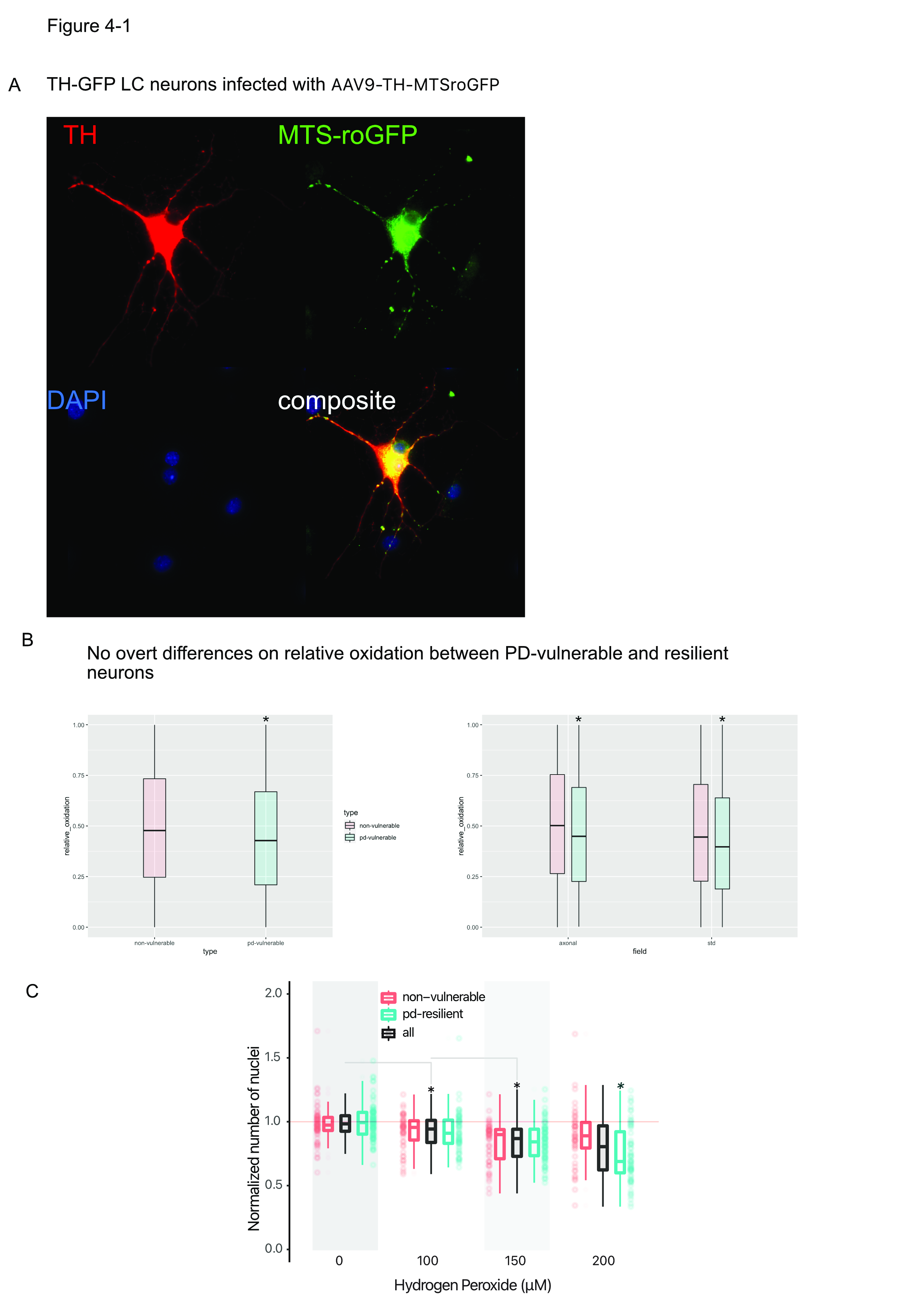

Supplement: Extended Data Figure 4-1 — A, Expression of MTSroGFP in locus coeruleus neurons. Photomicrographs of a LC noradrenergic neuron infected with AAV9-TH-MTSroGFP. The neuron is identified by the presence of TH (red). MTSroGFP is shown in green. Nuclei, stained with DAPI, are shown in blue. B, Comparing relative oxidation in neuron types and in somatodendritic domain and axons shows only very small differences. Compared with Welch two-sample t test. C, Normalized number of nuclei (DAPI-positive) across hydrogen peroxide concentrations. Box and whiskers plots, in the style of Tukey, where the median value is indicated, and the lower and upper hinges correspond to the first and third quartiles. * = one-way ANOVA, Tukey’s HSD test, p < 0.05; * = pairwise t test, vulnerable versus resilient, p < 0.05. Download Figure 4-1, TIF file. [file enu-eN-NWR-0139-22-s02.tif]
