# Supplementary material for: Axonal Domain Structure as a Putative Identifier of Neuron-Specific Vulnerability to Oxidative Stress in Cultured Neurons
Source: eNeuro. 2022 Oct 24;9(5):ENEURO.0139-22.2022. doi: 10.1523/ENEURO.0139-22.2022 (PMC9595591; doi:10.1523/ENEURO.0139-22.2022)
Supplement: Extended Data Table 4-1 — Statistical reporting for Figure 4C. Download Table 4-1, DOCX file. [file enu-eN-NWR-0139-22-s05.docx]

**EXTENDED TABLES FOR FIGURE 4C**

Kruskal-Wallis

Kruskal-Wallis rank sum test

data: relative_oxidation by neuron
Kruskal-Wallis chi-squared = 133.58, df = 6, p-value < 2.2e-16

Dunn

| Comparison | Z | P.unadj | P.adj |
| --- | --- | --- | --- |
| DMV - LC | -3.3231328 | 0.0008901 | 0.0186926 |
| DMV - R | -7.8362467 | 0.0000000 | 0.0000000 |
| LC - R | -2.3435415 | 0.0191016 | 0.4011343 |
| DMV - SN | -5.8719183 | 0.0000000 | 0.0000001 |
| LC - SN | -0.2064038 | 0.8364755 | 1.0000000 |
| R - SN | 2.8602152 | 0.0042335 | 0.0889043 |
| DMV - STR | -7.9501041 | 0.0000000 | 0.0000000 |
| LC - STR | -0.3988749 | 0.6899854 | 1.0000000 |
| R - STR | 2.9232967 | 0.0034635 | 0.0727327 |
| SN - STR | -0.2802927 | 0.7792529 | 1.0000000 |
| DMV - VTA | -5.1989318 | 0.0000002 | 0.0000042 |
| LC - VTA | 0.9733339 | 0.3303874 | 1.0000000 |
| R - VTA | 4.6926942 | 0.0000027 | 0.0000566 |
| SN - VTA | 1.8997528 | 0.0574656 | 1.0000000 |
| STR - VTA | 2.7090509 | 0.0067476 | 0.1416996 |
| DMV - XII | -7.9440379 | 0.0000000 | 0.0000000 |
| LC - XII | -0.5001405 | 0.6169762 | 1.0000000 |
| R - XII | 2.7666535 | 0.0056635 | 0.1189333 |
| SN - XII | -0.4371405 | 0.6620094 | 1.0000000 |
| STR - XII | -0.1955555 | 0.8449581 | 1.0000000 |
| VTA - XII | -2.8474318 | 0.0044074 | 0.0925544 |

**Estimation statistics**

| control_group | test_group | difference | bca_ci_low | bca_ci_high |
| --- | --- | --- | --- | --- |
| SN | VTA | -0.028 | -0.055 | 0.001 |
| SN | LC | -0.004 | -0.052 | 0.041 |
| SN | R | 0.059 | 0.018 | 0.099 |
| SN | DMV | -0.078 | -0.103 | -0.052 |
| SN | XII | 0.007 | -0.023 | 0.035 |
| SN | STR | 0.005 | -0.024 | 0.033 |
