# Supplementary material for: Axonal Domain Structure as a Putative Identifier of Neuron-Specific Vulnerability to Oxidative Stress in Cultured Neurons
Source: eNeuro. 2022 Oct 24;9(5):ENEURO.0139-22.2022. doi: 10.1523/ENEURO.0139-22.2022 (PMC9595591; doi:10.1523/ENEURO.0139-22.2022)
Supplement: Extended Data Table 4-2 — Statistical reporting for Figure 4D. Download Table 4-2, DOCX file. [file enu-eN-NWR-0139-22-s06.docx]

**EXTENDED TABLE FOR FIGURE 4D**

Kurskal-Wallis

Kruskal-Wallis rank sum test

data: distance_um by neuron
Kruskal-Wallis chi-squared = 1.4927, df = 6, p-value = 0.96

Estimation statistics — not applicable
