# Supplementary material for: Axonal Domain Structure as a Putative Identifier of Neuron-Specific Vulnerability to Oxidative Stress in Cultured Neurons
Source: eNeuro. 2022 Oct 24;9(5):ENEURO.0139-22.2022. doi: 10.1523/ENEURO.0139-22.2022 (PMC9595591; doi:10.1523/ENEURO.0139-22.2022)
Supplement: Extended Data Table 6-1 — Statistical reporting for Figure 6B–D. Download Table 6-1, DOCX file. [file enu-eN-NWR-0139-22-s07.docx]

**EXTENDED TABLES FOR FIGURE 6B**

Kruskal-Wallis

Kruskal-Wallis rank sum test

data: axon_area_per_neuron by neuron
Kruskal-Wallis chi-squared = 49.602, df = 6, p-value = 5.649e-09

Dunn

| Comparison | Z | P.unadj | P.adj |
| --- | --- | --- | --- |
| DMV - LC | -1.4910572 | 0.1359465 | 1.0000000 |
| DMV - R | -2.1697753 | 0.0300239 | 0.6305013 |
| LC - R | -0.6949384 | 0.4870939 | 1.0000000 |
| DMV - SN | 0.3799178 | 0.7040065 | 1.0000000 |
| LC - SN | 1.7261794 | 0.0843151 | 1.0000000 |
| R - SN | 2.3506380 | 0.0187413 | 0.3935664 |
| DMV - STR | 1.8240271 | 0.0681480 | 1.0000000 |
| LC - STR | 2.9510478 | 0.0031670 | 0.0665066 |
| R - STR | 3.4823377 | 0.0004971 | 0.0104382 |
| SN - STR | 1.3978009 | 0.1621729 | 1.0000000 |
| DMV - VTA | 4.2860413 | 0.0000182 | 0.0003820 |
| LC - VTA | 5.3781860 | 0.0000001 | 0.0000016 |
| R - VTA | 5.8726242 | 0.0000000 | 0.0000001 |
| SN - VTA | 3.5891017 | 0.0003318 | 0.0069682 |
| STR - VTA | 1.7920665 | 0.0731223 | 1.0000000 |
| DMV - XII | 1.8995788 | 0.0574884 | 1.0000000 |
| LC - XII | 2.8822979 | 0.0039479 | 0.0829051 |
| R - XII | 3.3562827 | 0.0007900 | 0.0165895 |
| SN - XII | 1.5407671 | 0.1233735 | 1.0000000 |
| STR - XII | 0.3079467 | 0.7581229 | 1.0000000 |
| VTA - XII | -1.2158840 | 0.2240291 | 1.0000000 |

**Estimation statistics**

| control_group | test_group | difference | bca_ci_low | bca_ci_high |
| --- | --- | --- | --- | --- |
| SN | VTA | -9453.439 | -16873.614 | -5078.015 |
| SN | LC | 594.818 | -7337.320 | 5639.899 |
| SN | R | 2921.211 | -4572.159 | 8595.133 |
| SN | DMV | -1285.267 | -8569.333 | 4096.503 |
| SN | XII | -6943.180 | -15300.619 | -1848.567 |
| SN | STR | -5664.433 | -13692.202 | -190.316 |

*PD-vulnerable vs PD-resilient*

Wilcoxon rank sum test with continuity correction

data: data$axon_area_per_neuron by data$type
W = 723, p-value = 6.78e-10
alternative hypothesis: true location shift is not equal to 0

**EXTENDED TABLES FOR FIGURE 6C**

Kruskal-Wallis

Kruskal-Wallis rank sum test

data: count_per_neuron by neuron
Kruskal-Wallis chi-squared = 67.386, df = 6, p-value = 1.403e-12

Dunn

| Comparison | Z | P.unadj | P.adj |
| --- | --- | --- | --- |
| DMV - LC | -2.1117441 | 0.0347084 | 0.7288765 |
| DMV - R | -4.9124144 | 0.0000009 | 0.0000189 |
| LC - R | -2.7062408 | 0.0068050 | 0.1429043 |
| DMV - SN | -2.8158643 | 0.0048646 | 0.1021570 |
| LC - SN | -0.6831796 | 0.4944934 | 1.0000000 |
| R - SN | 2.0185955 | 0.0435293 | 0.9141149 |
| DMV - STR | -2.4474832 | 0.0143858 | 0.3021014 |
| LC - STR | -0.5833195 | 0.5596782 | 1.0000000 |
| R - STR | 1.8401285 | 0.0657494 | 1.0000000 |
| SN - STR | 0.0260784 | 0.9791948 | 1.0000000 |
| DMV - VTA | 2.7269895 | 0.0063915 | 0.1342216 |
| LC - VTA | 4.5046159 | 0.0000066 | 0.0001396 |
| R - VTA | 7.0429233 | 0.0000000 | 0.0000000 |
| SN - VTA | 5.1351081 | 0.0000003 | 0.0000059 |
| STR - VTA | 4.5560173 | 0.0000052 | 0.0001095 |
| DMV - XII | 1.1254594 | 0.2603944 | 1.0000000 |
| LC - XII | 2.5790534 | 0.0099071 | 0.2080501 |
| R - XII | 4.6164416 | 0.0000039 | 0.0000820 |
| SN - XII | 3.0845492 | 0.0020386 | 0.0428108 |
| STR - XII | 2.8552967 | 0.0042997 | 0.0902929 |
| VTA - XII | -0.8534673 | 0.3934002 | 1.0000000 |

**Estimation statistics**

| control_group | test_group | difference | bca_ci_low | bca_ci_high |
| --- | --- | --- | --- | --- |
| SN | VTA | -480.637 | -647.429 | -330.347 |
| SN | LC | -146.549 | -328.531 | 30.970 |
| SN | R | 280.998 | 73.514 | 530.019 |
| SN | DMV | -253.894 | -443.260 | -42.919 |
| SN | XII | -417.482 | -595.935 | -259.965 |
| SN | STR | 43.933 | -221.949 | 374.863 |

*PD-vulnerable vs PD-resilient*

Wilcoxon rank sum test with continuity correction

data: data$count_per_neuron by data$type
W = 1068, p-value = 4.433e-06
alternative hypothesis: true location shift is not equal to 0

**EXTENDED TABLES FOR FIGURE 6D**

Average segmentation length (µm)

Kruskal-Wallis

Kruskal-Wallis rank sum test

data: Average Size by neuron
Kruskal-Wallis chi-squared = 63.024, df = 6, p-value = 1.092e-11

Dunn

| Comparison | Z | P.unadj | P.adj |
| --- | --- | --- | --- |
| DMV - LC | 1.0305973 | 0.3027297 | 1.0000000 |
| DMV - R | 3.6099861 | 0.0003062 | 0.0064305 |
| LC - R | 2.4559112 | 0.0140528 | 0.2951085 |
| DMV - SN | 6.3579580 | 0.0000000 | 0.0000000 |
| LC - SN | 4.9813038 | 0.0000006 | 0.0000133 |
| R - SN | 2.3704309 | 0.0177674 | 0.3731146 |
| DMV - STR | 5.3220750 | 0.0000001 | 0.0000022 |
| LC - STR | 4.1924230 | 0.0000276 | 0.0005796 |
| R - STR | 1.8856112 | 0.0593474 | 1.0000000 |
| SN - STR | -0.2503577 | 0.8023108 | 1.0000000 |
| DMV - VTA | 1.8588286 | 0.0630514 | 1.0000000 |
| LC - VTA | 0.7710414 | 0.4406824 | 1.0000000 |
| R - VTA | -1.7136144 | 0.0865996 | 1.0000000 |
| SN - VTA | -4.2192807 | 0.0000245 | 0.0005147 |
| STR - VTA | -3.5124283 | 0.0004440 | 0.0093247 |
| DMV - XII | 0.8190679 | 0.4127477 | 1.0000000 |
| LC - XII | 0.0556341 | 0.9556333 | 1.0000000 |
| R - XII | -1.8463948 | 0.0648349 | 1.0000000 |
| SN - XII | -3.7591493 | 0.0001705 | 0.0035803 |
| STR - XII | -3.3032427 | 0.0009557 | 0.0200705 |
| VTA - XII | -0.5319001 | 0.5947952 | 1.0000000 |

**Estimation statistics**

| control_group | test_group | difference | bca_ci_low | bca_ci_high |
| --- | --- | --- | --- | --- |
| SN | VTA | 64.612 | 31.380 | 99.114 |
| SN | LC | 35.757 | 15.428 | 52.260 |
| SN | R | 7.734 | -13.329 | 18.413 |
| SN | DMV | 77.256 | 50.864 | 103.509 |
| SN | XII | 39.640 | 13.643 | 59.692 |
| SN | STR | -5.698 | -27.024 | 5.072 |

*PD-vulnerable vs PD-resilient*

Wilcoxon rank sum test with continuity correction

data: data$`Average Size` by data$type
W = 1887, p-value = 0.4029
alternative hypothesis: true location shift is not equal to 0
