# Supplementary material for: Axonal Domain Structure as a Putative Identifier of Neuron-Specific Vulnerability to Oxidative Stress in Cultured Neurons
Source: eNeuro. 2022 Oct 24;9(5):ENEURO.0139-22.2022. doi: 10.1523/ENEURO.0139-22.2022 (PMC9595591; doi:10.1523/ENEURO.0139-22.2022)
Supplement: Extended Data Table 8-1 — Statistical reporting for Figure 8B–D. Download Table 8-1, DOCX file. [file enu-eN-NWR-0139-22-s08.docx]

**EXTENDED TABLES FOR FIGURE 8B**

Kruskal-Wallis

Kruskal-Wallis rank sum test

data: percentage_syt1_positive by neuron
Kruskal-Wallis chi-squared = 59.589, df = 6, p-value = 5.456e-11

Dunn

| Comparison | Z | P.unadj | P.adj |
| --- | --- | --- | --- |
| DMV - LC | 0.8368274 | 0.4026896 | 1.0000000 |
| DMV - R | -1.5062893 | 0.1319929 | 1.0000000 |
| LC - R | -2.3431167 | 0.0191234 | 0.4015914 |
| DMV - SN | -0.1422607 | 0.8868741 | 1.0000000 |
| LC - SN | -0.9790881 | 0.3275365 | 1.0000000 |
| R - SN | 1.3640287 | 0.1725585 | 1.0000000 |
| DMV - STR | 3.0042104 | 0.0026627 | 0.0559169 |
| LC - STR | 2.1673830 | 0.0302057 | 0.6343189 |
| R - STR | 4.5104997 | 0.0000065 | 0.0001358 |
| SN - STR | 3.1464711 | 0.0016525 | 0.0347033 |
| DMV - VTA | 3.1632076 | 0.0015604 | 0.0327686 |
| LC - VTA | 2.3263802 | 0.0199983 | 0.4199638 |
| R - VTA | 4.6694969 | 0.0000030 | 0.0000634 |
| SN - VTA | 3.3054683 | 0.0009482 | 0.0199117 |
| STR - VTA | 0.1589972 | 0.8736711 | 1.0000000 |
| DMV - XII | 4.7197066 | 0.0000024 | 0.0000496 |
| LC - XII | 3.8828792 | 0.0001032 | 0.0021678 |
| R - XII | 6.2259959 | 0.0000000 | 0.0000000 |
| SN - XII | 4.8619672 | 0.0000012 | 0.0000244 |
| STR - XII | 1.7154962 | 0.0862543 | 1.0000000 |
| VTA - XII | 1.5564990 | 0.1195895 | 1.0000000 |

**Estimation statistics**

| control_group | test_group | difference | bca_ci_low | bca_ci_high |
| --- | --- | --- | --- | --- |
| SN | VTA | -32.085 | -43.167 | -19.261 |
| SN | LC | -8.150 | -18.037 | 2.028 |
| SN | R | 11.295 | 1.790 | 20.876 |
| SN | DMV | -1.493 | -10.867 | 7.755 |
| SN | XII | -48.120 | -56.935 | -38.492 |
| SN | STR | -29.978 | -40.417 | -17.486 |

*PD-vulnerable vs PD-resilient*

Wilcoxon rank sum exact test

data: data_percentage_syt1_positive$percentage_syt1_positive by data_percentage_syt1_positive$type
W = 79, p-value = 2.941e-16
alternative hypothesis: true location shift is not equal to 0

**EXTENDED TABLES FOR FIGURE 8C**

Kruskal-Wallis

Kruskal-Wallis rank sum test

data: mean_nearest_varicsotiey by neuron
Kruskal-Wallis chi-squared = 28.368, df = 6, p-value = 8.011e-05

Dunn

| Comparison | Z | P.unadj | P.adj |
| --- | --- | --- | --- |
| DMV - LC | 0.8703005 | 0.3841362 | 1.0000000 |
| DMV - R | -0.5941475 | 0.5524135 | 1.0000000 |
| LC - R | -1.4644480 | 0.1430716 | 1.0000000 |
| DMV - SN | 3.1297345 | 0.0017496 | 0.0367425 |
| LC - SN | 2.2594340 | 0.0238564 | 0.5009844 |
| R - SN | 3.7238820 | 0.0001962 | 0.0041198 |
| DMV - STR | 1.5732355 | 0.1156643 | 1.0000000 |
| LC - STR | 0.7029350 | 0.4820962 | 1.0000000 |
| R - STR | 2.1673830 | 0.0302057 | 0.6343189 |
| SN - STR | -1.5564990 | 0.1195895 | 1.0000000 |
| DMV - VTA | 2.0334906 | 0.0420030 | 0.8820626 |
| LC - VTA | 1.1631901 | 0.2447524 | 1.0000000 |
| R - VTA | 2.6276381 | 0.0085980 | 0.1805579 |
| SN - VTA | -1.0962439 | 0.2729721 | 1.0000000 |
| STR - VTA | 0.4602551 | 0.6453331 | 1.0000000 |
| DMV - XII | -1.2133997 | 0.2249770 | 1.0000000 |
| LC - XII | -2.0837002 | 0.0371874 | 0.7809363 |
| R - XII | -0.6192523 | 0.5357502 | 1.0000000 |
| SN - XII | -4.3431343 | 0.0000140 | 0.0002950 |
| STR - XII | -2.7866353 | 0.0053258 | 0.1118426 |
| VTA - XII | -3.2468903 | 0.0011667 | 0.0245014 |

**Estimation Statistics**

| control_group | test_group | difference | bca_ci_low | bca_ci_high |
| --- | --- | --- | --- | --- |
| SN | VTA | 0.162 | -0.077 | 0.415 |
| SN | LC | 0.406 | 0.183 | 0.789 |
| SN | R | 0.871 | 0.507 | 1.500 |
| SN | DMV | 0.740 | 0.375 | 1.411 |
| SN | XII | 1.332 | 0.708 | 3.438 |
| SN | STR | 0.331 | 0.056 | 0.717 |

*PD-vulnerable vs PD-resilient*

Wilcoxon rank sum exact test

data: mean_varicosity_nn_length$mean_nearest_varicsotiey by mean_varicosity_nn_length$type
W = 875, p-value = 0.9248
alternative hypothesis: true location shift is not equal to 0

**EXTENDED TABLES FOR FIGURE 8D**

Kruskal-Wallis

Kruskal-Wallis rank sum test

data: mean_axon_per_var_px by neuron
Kruskal-Wallis chi-squared = 62.576, df = 6, p-value = 1.346e-11

Dunn

| Comparison | Z | P.unadj | P.adj |
| --- | --- | --- | --- |
| DMV - LC | 4.6611287 | 0.0000031 | 0.0000660 |
| DMV - R | 4.1506639 | 0.0000332 | 0.0006962 |
| LC - R | -0.5104647 | 0.6097259 | 1.0000000 |
| DMV - SN | 4.4519218 | 0.0000085 | 0.0001787 |
| LC - SN | -0.2092069 | 0.8342868 | 1.0000000 |
| R - SN | 0.3012579 | 0.7632179 | 1.0000000 |
| DMV - STR | 6.1255766 | 0.0000000 | 0.0000000 |
| LC - STR | 1.4644480 | 0.1430716 | 1.0000000 |
| R - STR | 1.9749127 | 0.0482781 | 1.0000000 |
| SN - STR | 1.6736548 | 0.0941985 | 1.0000000 |
| DMV - VTA | 2.5857967 | 0.0097154 | 0.2040239 |
| LC - VTA | -2.0753320 | 0.0379558 | 0.7970713 |
| R - VTA | -1.5648673 | 0.1176140 | 1.0000000 |
| SN - VTA | -1.8661251 | 0.0620239 | 1.0000000 |
| STR - VTA | -3.5397799 | 0.0004005 | 0.0084097 |
| DMV - XII | 0.4602551 | 0.6453331 | 1.0000000 |
| LC - XII | -4.2008736 | 0.0000266 | 0.0005584 |
| R - XII | -3.6904089 | 0.0002239 | 0.0047018 |
| SN - XII | -3.9916667 | 0.0000656 | 0.0013778 |
| STR - XII | -5.6653216 | 0.0000000 | 0.0000003 |
| VTA - XII | -2.1255416 | 0.0335414 | 0.7043703 |

**Estimation Statistics**

| control_group | test_group | difference | bca_ci_low | bca_ci_high |
| --- | --- | --- | --- | --- |
| SN | VTA | 7.053 | 3.235 | 13.312 |
| SN | LC | 0.197 | -3.298 | 4.452 |
| SN | R | 0.326 | -2.720 | 2.683 |
| SN | DMV | 45.600 | 34.165 | 72.217 |
| SN | XII | 33.246 | 24.793 | 47.406 |
| SN | STR | -3.624 | -6.413 | -1.893 |

*PD-vulnerable vs PD-resilient*

Wilcoxon rank sum exact test

data: mean_varicosity_lengthpervar_length$mean_axon_per_var_px by mean_varicosity_lengthpervar_length$type
W = 917, p-value = 0.6369
alternative hypothesis: true location shift is not equal to 0
